# Essential antiarrhythmic drug accessibility worldwide: a multi-survey study and comprehensive evaluation of access and supply challenges

**DOI:** 10.1093/europace/euag220

**Published:** 2026-09-02

**Authors:** Pieter G Postema, Niels D Reijnhout, Federico Ballacci, Evert A Manders, Wiert F Hoeksema, Mauro Toniolo, Jacob Tfelt-Hansen, Diego Penela, Piotr Futyma, Deirdre A Lane, Ruben Casado-Arroyo, Georgia Sarquella-Brugada, Nico A Blom, Arthur A M Wilde, Carla E M Hollak, Elena Arbelo

**Affiliations:** Department of Clinical and Experimental Cardiology, Heart Center, Amsterdam UMC, University of Amsterdam, Cardiovascular Sciences, Meibergdreef 9, Amsterdam 1105, The Netherlands; EHRA Headquarters, EHRA, European Heart House, Sophia Antipolis Cedex, France; European Reference Network (ERN) GUARD-Heart for rare and complex diseases of the heart, Amsterdam, The Netherlands; Medicine for Society Platform at Amsterdam University Medical Centers, University of Amsterdam, Amsterdam, The Netherlands; Cardiology Division, University Hospital “S. Maria della Misericordia”, Udine, Italy; Medicine for Society Platform at Amsterdam University Medical Centers, University of Amsterdam, Amsterdam, The Netherlands; Department of Endocrinology and Metabolism, Amsterdam University Medical Centers, University of Amsterdam, Amsterdam, The Netherlands; Department of Clinical and Experimental Cardiology, Heart Center, Amsterdam UMC, University of Amsterdam, Cardiovascular Sciences, Meibergdreef 9, Amsterdam 1105, The Netherlands; EHRA Headquarters, EHRA, European Heart House, Sophia Antipolis Cedex, France; European Reference Network (ERN) GUARD-Heart for rare and complex diseases of the heart, Amsterdam, The Netherlands; Cardiology Division, University Hospital “S. Maria della Misericordia”, Udine, Italy; European Reference Network (ERN) GUARD-Heart for rare and complex diseases of the heart, Amsterdam, The Netherlands; Department of Cardiology, Heart Centre, Copenhagen University Hospital, Rigshospitalet, Copenhagen, Denmark; Department of Forensic Medicine, Faculty of Medical Sciences, University of Copenhagen, Copenhagen, Denmark; Arrhythmia Section, Cardiology Department, Hospital Clinic, Universitat de Barcelona, Barcelona, Spain; St. Joseph's Heart Rhythm Center, University of Rzeszów, Rzeszów, Poland; EHRA Headquarters, EHRA, European Heart House, Sophia Antipolis Cedex, France; Department of Cardiovascular and Metabolic Medicine, Institute of Life Course and Medical Sciences, University of Liverpool, Liverpool, UK; Liverpool Centre for Cardiovascular Sciences, University of Liverpool, Liverpool John Moores University and Liverpool Heart and Chest Hospital, Liverpool, UK; EHRA Headquarters, EHRA, European Heart House, Sophia Antipolis Cedex, France; Department of Cardiology, Erasme University Hospital, Université Libre de Bruxelles, Brussels, Belgium; EHRA Headquarters, EHRA, European Heart House, Sophia Antipolis Cedex, France; European Reference Network (ERN) GUARD-Heart for rare and complex diseases of the heart, Amsterdam, The Netherlands; Arrhythmias, Inherited Cardiac Diseases and Sudden Death Unit, Hospital Sant Joan de Déu, Esplugues de Llobregat, Barcelona, Spain; Association for European Paediatric and Congenital Cardiology (AEPC), Geneva, Switzerland; European Reference Network (ERN) GUARD-Heart for rare and complex diseases of the heart, Amsterdam, The Netherlands; Association for European Paediatric and Congenital Cardiology (AEPC), Geneva, Switzerland; Department of Pediatric Cardiology, Amsterdam UMC, University of Amsterdam, Emma Children’s Hospital, Amsterdam, The Netherlands; Department of Clinical and Experimental Cardiology, Heart Center, Amsterdam UMC, University of Amsterdam, Cardiovascular Sciences, Meibergdreef 9, Amsterdam 1105, The Netherlands; European Reference Network (ERN) GUARD-Heart for rare and complex diseases of the heart, Amsterdam, The Netherlands; Medicine for Society Platform at Amsterdam University Medical Centers, University of Amsterdam, Amsterdam, The Netherlands; Department of Endocrinology and Metabolism, Amsterdam University Medical Centers, University of Amsterdam, Amsterdam, The Netherlands; EHRA Headquarters, EHRA, European Heart House, Sophia Antipolis Cedex, France; European Reference Network (ERN) GUARD-Heart for rare and complex diseases of the heart, Amsterdam, The Netherlands; Institut d’Investigacio August Pi i Sunyer (IDIBAPS), Barcelona, Spain; Centro de Investigaci on Biome´dica en Red de Enfermedades Cardiovasculares (CIBERCV), Madrid, Spain

**Keywords:** Antiarrhythmic drugs, Drug accessibility, Medicines shortages, Arrhythmia care, Survey

## Abstract

**Aims:**

Antiarrhythmic drugs (AADs) are essential for preventing symptoms, morbidity, and mortality associated with cardiac arrhythmias. However, concerns are increasing about their accessibility. We aimed to assess insights into current access to AADs.

**Methods and results:**

Online surveys were distributed to members of the European Heart Rhythm Association, the Association for European Paediatric and Congenital Cardiology, the European Reference Network GUARD-Heart, and national heart rhythm societies. Responses were obtained from 558 respondents across 80 countries. Among countries included in the main analysis (≥5 respondents; 16 countries within and 7 outside the European Union, *n* = 298 respondents), only 16–59% of surveyed AADs were considered readily accessible. Accessibility varied substantially by drug: oral flecainide and propranolol were available in 88% of countries, whereas mexiletine solution was available in only 10%. Marked differences in accessibility were reported both between and within countries, with availability also changing over time. Higher-income countries generally reported better access and a greater number of market authorization holders than lower-income countries. National societies’ perception was that limited access negatively affected patients’ quality of life (72%) and life expectancy (35%), while 73% expected these challenges to persist or worsen in the coming years.

**Conclusion:**

Physicians worldwide frequently encounter problems accessing guideline-recommended antiarrhythmic drugs for their patients, which putatively impacts their patients’ quality of life and life expectancy. This highlights systemic vulnerabilities that extend across national and institutional boundaries, urging close collaboration among clinicians, policymakers, manufacturers, and institutions to guarantee timely and equitable treatment for all patients.

## Graphical Abstract

**Graphical Abstract:**
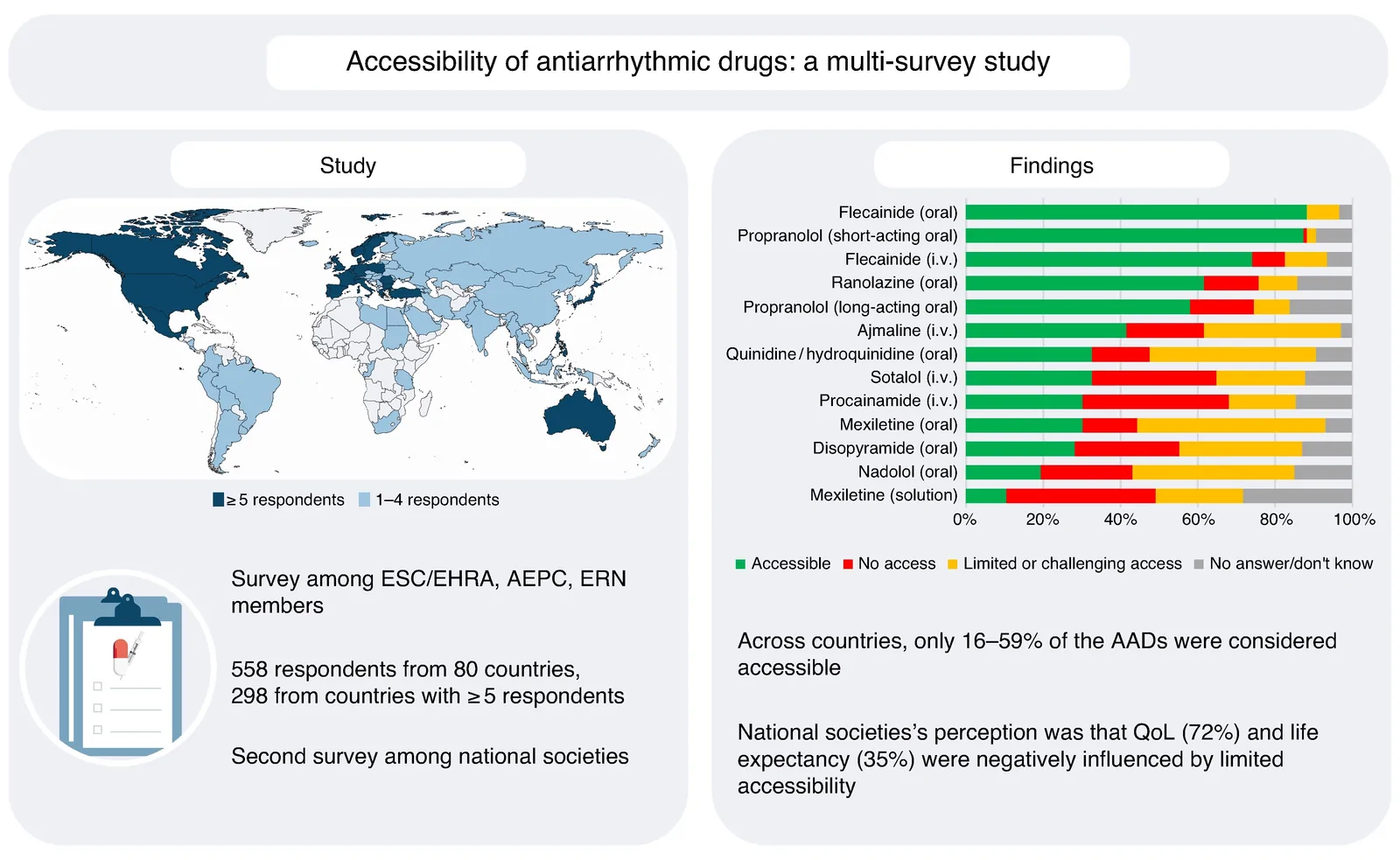

What’s new?

1. Multiple antiarrhythmic drugs, recommended in international guidelines, are not readily accessible in any country worldwide, with country-level coverage ranging from 16 to 59% of surveyed drugs.
2. Striking differences exist between and within countries regarding antiarrhythmic drug accessibility.
3. Lower reported antiarrhythmic accessibility appeared to coincide with fewer market authorization holders and lower national income, albeit that no formal association analyses could be performed on these data.
4. National societies perceived these accessibility problems to affect patients’ quality of life and, in some cases, life expectancy, highlighting persistent systemic vulnerabilities in arrhythmia care.

## Introduction

**Figure 1:**
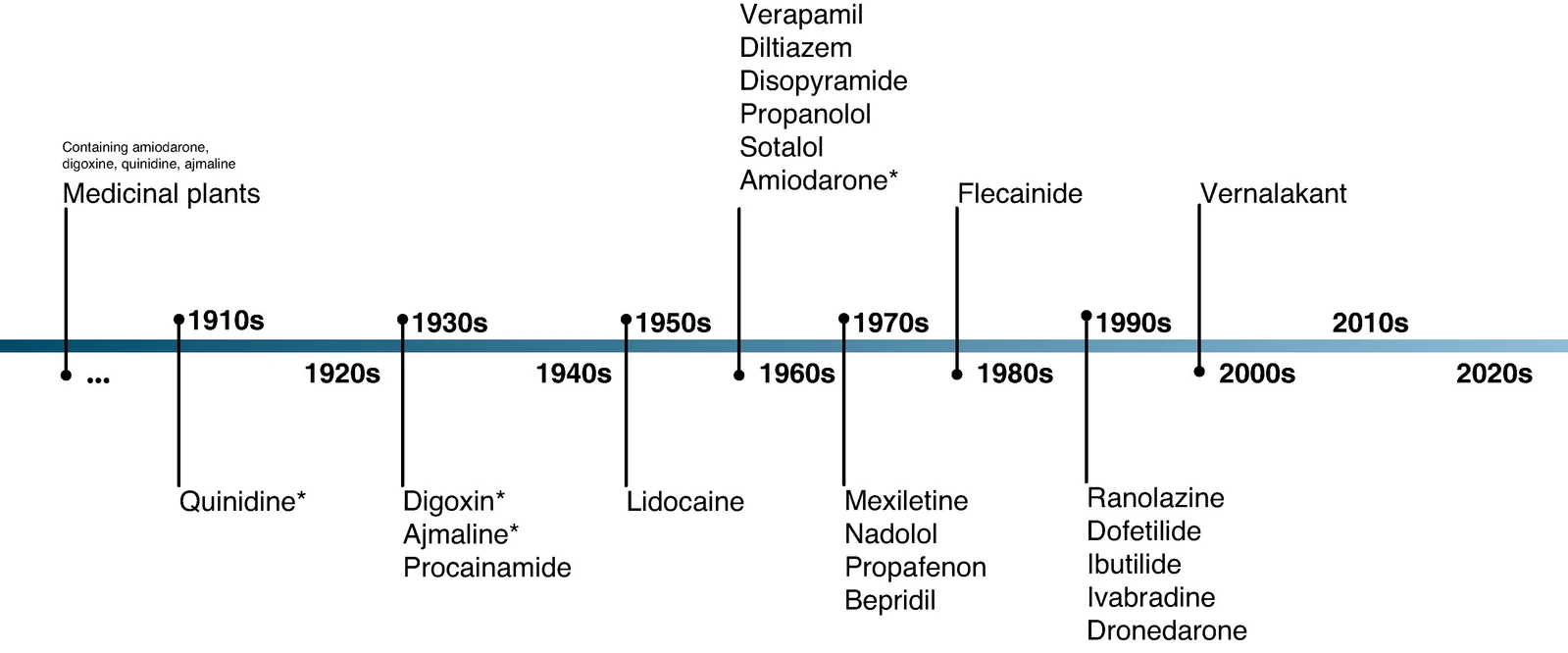
The recognition and development of antiarrhythmic drugs throughout history. Medicinal plants have been used for many centuries, while their specific active ingredients were developed in the 20th century (*). Some drugs have been designated as antiarrhythmic drugs many years after their development (e.g. ranolazine).

Antiarrhythmic drugs remain essential for the prevention and treatment of cardiac arrhythmias, improving symptoms and, in selected cases, reducing morbidity and mortality. The choice of a certain antiarrhythmic drug is dependent on the specific arrhythmia, the underlying mechanisms resulting in the arrhythmia, its burden and prognosis, comorbidities (such as structural heart disease and heart, kidney, and liver function), tolerability, and, equally important, accessibility. The development of nearly all antiarrhythmic drugs dates to the 1960s, 1970s, and 1990s (*Figure 1*). Over the last 30 years, there has been sparse development in this field, with later examples being ivabradine (1994),^1^ dronedarone (1995),^2^ and vernakalant (2007).^3^ The number of patients with an arrhythmia has increased exponentially in the past decades, causing sharp initial increases in antiarrhythmic drug use but with successive declines over time in the past years in several regions, putatively due to the limited accessibility of antiarrhythmic drugs.^4–7^ Thus, there is a pressing need to improve access to these drugs on a global scale. Antiarrhythmic drugs often have limited options for substitution by other drugs, increasing the implications of accessibility problems. If substitution is available, strict guidance and monitoring are required for successful application.

Accessibility has several dimensions, including regulatory approval, commercial availability, reimbursement, local formulary restrictions, affordability, and (temporary) shortages, which may differ considerably between and even within countries. Availability and affordability are the most important.^8^ For antiarrhythmic drugs, these dimensions are particularly important because limitations in access may affect both acute and long-term management across a broad range of arrhythmias.

Well-recognized accessibility examples include quinidine and mexiletine to treat malignant arrhythmia, illustrating how access problems arise when clinically important drugs become commercially fragile, difficult to source, or poorly supported by existing regulatory and reimbursement pathways.^9–11^ From these and other examples, multiple causes of these accessibility issues emerged, two major causes being an increased dependency on fewer supply chains and withdrawal of products from the market due to commercial reasons.^12,11,13^ In parallel, concerns about pro-arrhythmic risk and the substantial cost and complexity of drug development may have discouraged further innovation in antiarrhythmic therapy.^14–17^

To evaluate accessibility issues and to investigate the future perspective regarding antiarrhythmic drugs in Europe and beyond, the Advocacy, Quality Improvement and Health Economics Committee (AQIHEC) of the European Heart Rhythm Association (EHRA) and the Association for European Paediatric and Congenital Cardiology (AEPC), together with the European Society of Cardiology (ESC) and the European Reference Network (ERN) GUARD-Heart for rare and complex diseases of the heart, launched a survey amongst their members. In this survey, we aimed to establish a current view of the extent of problems that exist for physicians to acquire antiarrhythmic drugs for their patients and to explore factors influencing accessibility. These antiarrhythmic drugs cover both orphan drugs used to treat selected patients at risk for sudden death by specific malignant arrhythmia syndromes (who can be totally dependent on a single active ingredient, e.g. quinidine), as well as antiarrhythmic drugs used for very common but more benign arrhythmias such as atrial fibrillation.

## Methods

### Study design and survey development

This study was based on successive international online surveys designed to assess physician-reported accessibility of antiarrhythmic drugs, reflecting whether respondents considered a given drug obtainable for patient care within their practice setting, and to explore variation in access within and across countries. The survey instrument was developed iteratively with input from the EHRA-AQIHEC, AEPC, ESC, and the ERN GUARD-Heart. After evaluation and permission by the appropriate ESC-EHRA, AEPC and ERN GUARD-Heart bodies, similar to previous ESC-EHRA surveys,^18^ the survey was designed online using SurveyMonkey with support of the ESC-EHRA staff (see the Supplement). Notably, to adhere to the General Data Protection Regulation, the survey was designed to only capture anonymous data. A link to the survey was distributed through email to EHRA, AEPC, and ERN GUARD-Heart members and peer networks.

For the main analysis, the writing group selected antiarrhythmic drugs considered clinically relevant and potentially vulnerable to accessibility problems. The main analyses were subsequently limited to the following drugs: mexiletine (oral and suspension), nadolol (oral), propranolol (oral short-acting and long-acting), ajmaline (i.v.), procainamide (i.v.), disopyramide (oral), sotalol (i.v.), flecainide (oral and i.v.), and ranolazine (oral). Data regarding the other drugs are provided in the Supplementary materials.

### Survey analyses

The respondents were characterized based on country, primary workplace, profession, career stage, patient category (adult/paediatric), and experience in treatment decision-making regarding different arrhythmias. Respondents who did not answer any of the questions regarding accessibility of specific drugs were excluded. For the analysis, we included only countries with five or more respondents. Countries with less than five respondents were only categorized but not further investigated in detail to pertain a reasonable minimal standard for adequate data collection. While EHRA member countries also cover parts of Northern Africa and Western Asia, and individual EHRA members can be located all over the world, there is significant influence of the European Union (EU) on national legislation and regulation on drug accessibility. Therefore, we evaluated responses from countries within the EU (including Norway, Switzerland, and the UK, which comply largely with EU regulation) separately from responses from countries outside the EU (e.g. Australia, Canada, Japan, Mexico, Philippines, Turkey, and the USA).

In an extended accessibility analysis limited to EU countries plus the UK, we explored the number of marketing authorization holders for the active substances of these drugs per country as a potential factor influencing accessibility. Data were obtained from the EU Article 57 database, the repository of all medicines authorized in the European Economic Area, with additional investigations for the UK.

### Follow-up consultation with national societies

To validate and expand on these results, we sent out a summary of the national results of the initial survey to the national (heart rhythm) societies asking for their reflections. This was done for the EHRA countries with any number of respondents and for the countries outside EHRA with ≥5 respondents. With this additional round of characterization, we aimed to strengthen the respondents’ perceived availability issues, to possibly gain more insights into the national regulatory affairs that may hamper availability, and to collect a reflection in a different time frame (about a year later) to see whether availability issues change over time. This round of evaluations was submitted in the first half of 2025 and 2026.

## Results

### Respondent characteristics and survey coverage

The survey was distributed at the end of 2023 through mailings, social media, and peer networks. It attracted 558 respondents, mainly from Europe, but also from the Americas, Africa, Asia, and Oceania. Most respondents were male (66%), between 40 and 49 years of age (30%), working in academia (64%), in cardiac electrophysiology (59%), treating adults (75%), children (8%), or both (17%), and making daily or weekly treatment decisions for atrial fibrillation (80%), ventricular tachycardia (63%), and rare or inheritable arrhythmia syndromes (32%).

**Figure 2:**
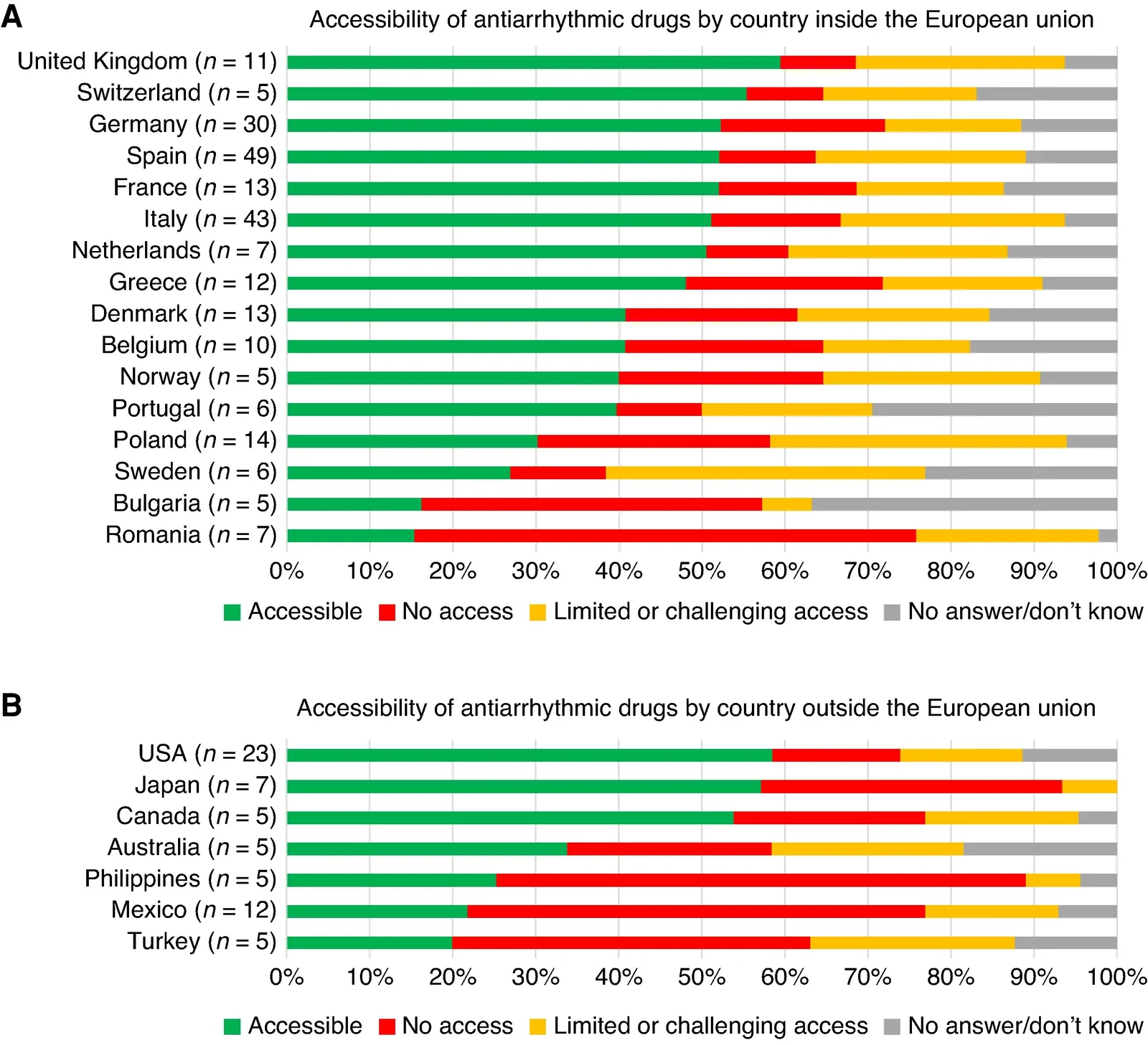
Reported accessibility of surveyed antiarrhythmic drugs by country grouping among countries with ≥5 respondents. (*A*) Reported accessibility of surveyed antiarrhythmic drugs by country within the European Union (plus Norway, Switzerland, and the UK) with ≥5 respondents. (*B*) Accessibility of surveyed antiarrhythmic drugs by country outside the European Union with ≥5 respondents.

**Figure 3:**
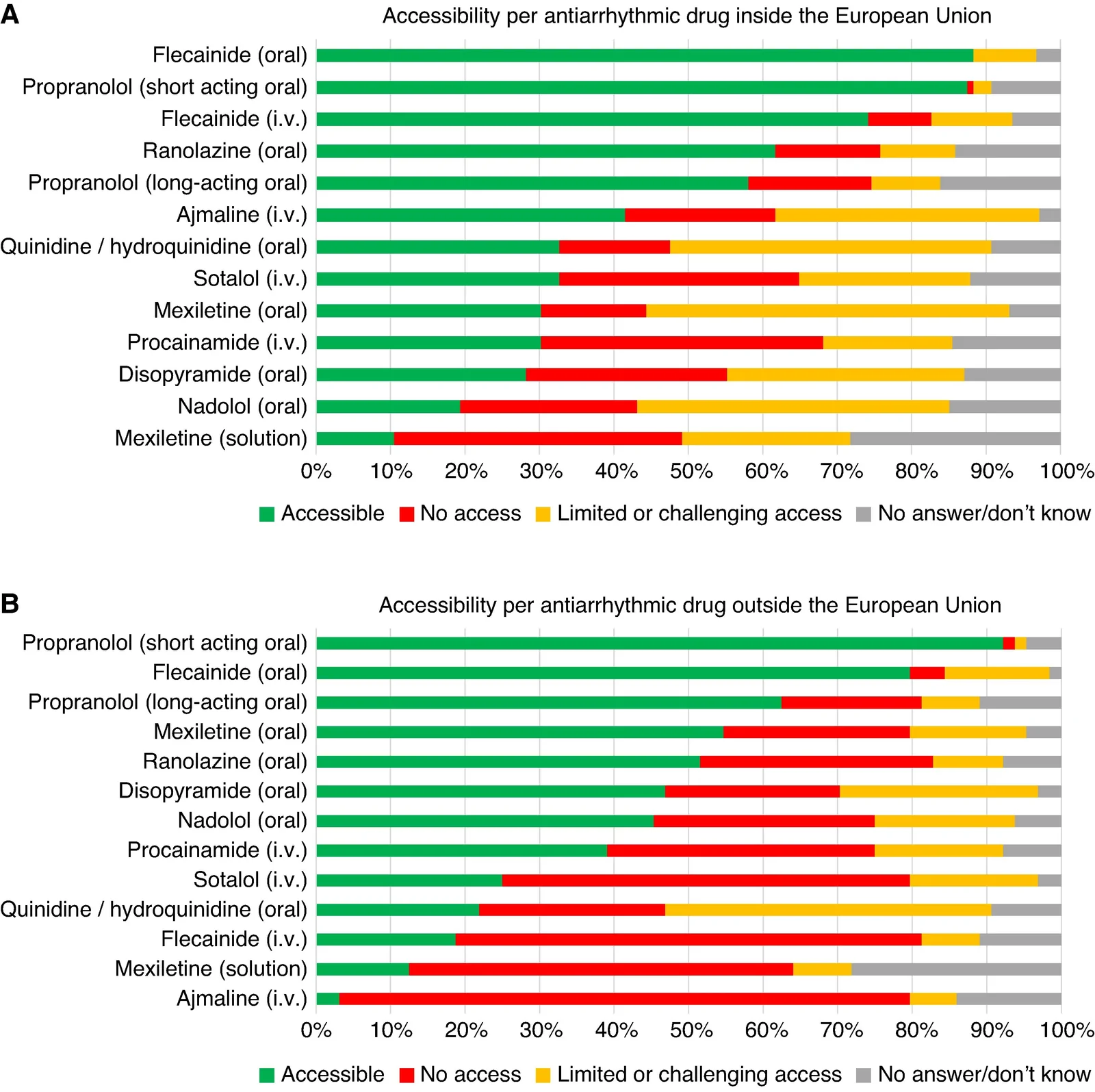
Reported accessibility of individual antiarrhythmic drugs, ranked by country grouping among countries with ≥5 respondents. (*A*) Accessibility of individual antiarrhythmic drugs (ranked) across countries in the European Union (plus Norway, Switzerland, and the UK) with ≥5 respondents. (*B*) Accessibility of individual antiarrhythmic drugs (ranked) across countries outside the European Union with ≥5 respondents.

In total, we received responses from 43 out of 55 EHRA member countries (78%) and from 37 countries outside EHRA member countries. An overview of the accessibility of all antiarrhythmic drugs reported in this survey for countries with ≥5 respondents (16 countries within and 7 outside the EU, *n* = 298 respondents) is presented in *Figure 2*, separated by countries within the EU (A) and outside the EU (B), while *Figure 3A* and *B* illustrate the accessibility of individual products from the main analysis within these countries.

Although the response rate to this survey ranks among the highest for ESC surveys, the wide range of respondents and divergent results meant that country-level analyses, even within the EU, were limited by small sample sizes. Nevertheless, several observations could be made.

### Accessibility across countries and drugs

The accessibility of antiarrhythmic drugs varied substantially across countries inside and outside the EU. For the EU, overall, Romania showed the lowest accessibility, with only 16% of the assessed products being readily accessible, whereas the UK demonstrated the highest accessibility, with 59% of products being reported as readily accessible (*Figure 2A*). Accessibility tended to be higher in countries with greater national income compared with those with lower income levels within the EU (*Figure 2*), albeit that no formal association analyses could be performed on these data. Sweden appeared to be an outlier in this regard, with respondents declaring relatively limited accessibility despite its high national income.^19^

Outside the EU, overall, Turkey showed the lowest reported availability, with only 20% of the assessed products considered readily accessible, whereas the USA demonstrated the highest accessibility, with 59% of products being reported as readily accessible (*Figure 2B*).

Regarding individual products, oral flecainide and propranolol were the most widely available both inside and outside the EU, reported as readily accessible in 88% of countries. In contrast, mexiletine (solution) was readily accessible in only about 10–12% of countries inside and outside the EU, making it one of the least accessible antiarrhythmic drugs together with ajmaline (3%) and i.v. flecainide (19%) outside the EU (*Figure 3A* and *B*). More data on the other surveyed AAD inside and outside the EU are available from the Supplementary material online, *Figures S1*–*S3*.

### Variability within countries and over time

Some antiarrhythmic drugs show accessibility issues across different countries—suggesting that some antiarrhythmic drugs have broad cross-national accessibility issues. Examples are mexiletine, nadolol, and procainamide. However, there is high variability in accessibility; while certain antiarrhythmic drugs are accessible in one country, they are not accessible in another country, and vice versa. An example of this pattern is ranolazine (see Supplementary material online, *Figure S2*). In some countries, however, accessibility issues for certain antiarrhythmic drugs are (very) differently perceived, suggesting that some physicians have access, but others do not or experience difficulties obtaining the drugs. Examples hereof are disopyramide, sotalol, and procainamide in multiple countries.

### Exploratory analysis of market authorization holders

**Figure 4:**
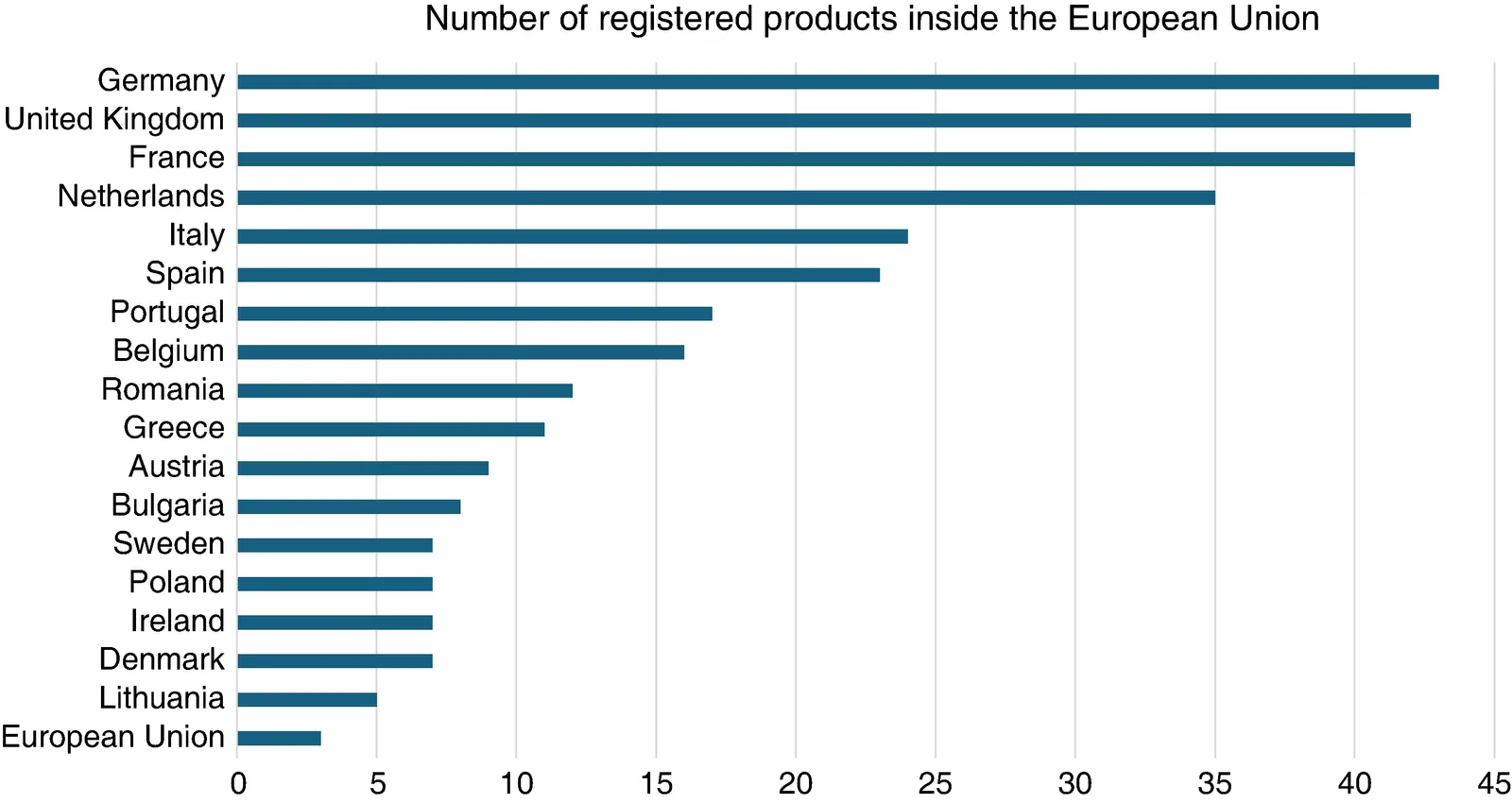
Number of registered products inside the European Union. Number of registered products in countries with ≥5 respondents (limited to countries in the European Union, plus the UK). Note that companies can also register drugs in the ‘European Union’ in contrast to (separate) country-level registration.

**Figure 5:**
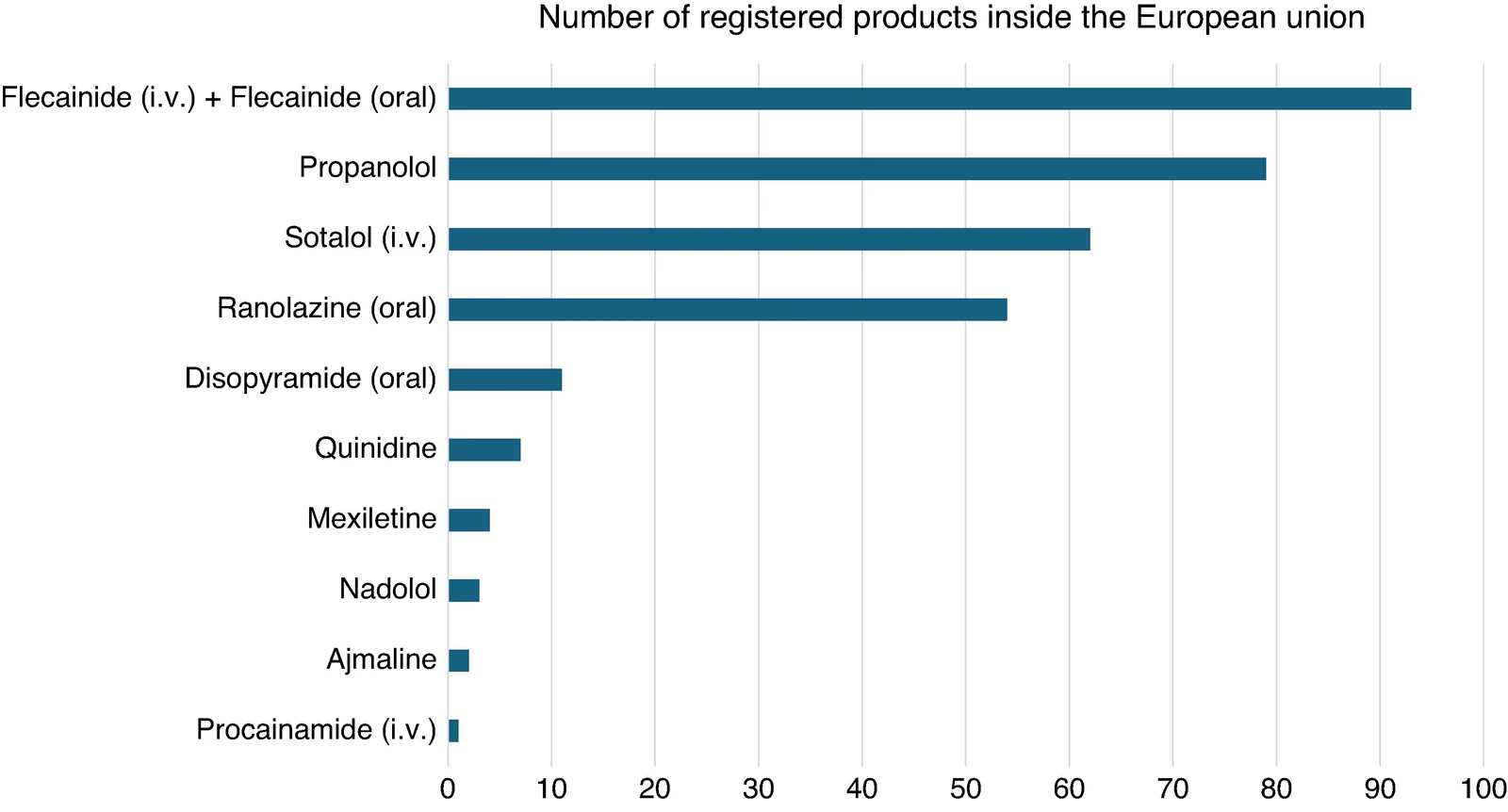
Number of registered antiarrhythmic drug products per active ingredient inside the European Union. Number of registered antiarrhythmic drug products per active substance (limited to countries in the European Union, plus the UK).

After an extended accessibility analysis of these results (limited to EU countries plus the UK), the following additional observations could be made:

1. Reported accessibility appeared to be greater in countries with higher numbers of registered products for the relevant active ingredient (i.e. a certain antiarrhythmic drug/active ingredient may be registered by 20 companies in one country but only one in another country). For example, France, Germany, and the UK have relatively high numbers of registered products (≥35) and reported fewer accessibility issues.
2. There is a marked difference in the number of registered products across different drugs. Propranolol, flecainide, sotalol, and ranolazine have over 50 market authorizations in the EU member states, while all others have 11 or fewer.
3. If antiarrhythmic drugs have significant accessibility issues, it appears that the number of registered drugs is low. Illustrations of these differences are depicted in *Figures 4* and *5*.

Additional insights in the survey results, including from the countries with <5 respondents and outside the EU, are available in the figures in the supplement. Also, barriers and solutions as perceived by the respondents, as well as data on coverage of the costs associated with the surveyed drugs, can be found in the supplements (see Supplementary material online, *Figures S1*–*S5*).

### Perception by national (heart rhythm) societies

From 41 of the 43 EHRA member countries with respondents to the initial survey (Russia and Belarus are currently suspended from the ESC and were thus not contacted), we finally received responses from 34 (83%) national (heart rhythm) societies to the summaries of their country results. Of the six countries with ≥5 respondents outside EHRA member countries, we received five responses (86%). On average, 71% agreed or strongly agreed with the outcomes of the initial survey on a per-drug basis; however, 22% disagreed or strongly disagreed. Remarks provided to these disagreements mirrored the general observations mentioned above for the initial survey, namely the divergence of accessibility. While some antiarrhythmic drugs were considered easily accessible by the individual respondents, a year later, they were hard to acquire in the views of the societies, and vice versa (for example, nadolol, mexiletine, and ranolazine). In addition, even antiarrhythmic drugs previously regarded as reasonably accessible (and not included in the main survey results, such as oral amiodarone and oral sotalol) were mentioned to have problems with accessibility (see Supplementary material online, *Figures S3A* and *B*). Arguably, this may in part be due to different time frames in which these surveys were gathered, making them prone to disruption or improvement in accessibility between the surveys. In addition, 72% and 35% of the societies feel that accessibility issues negatively impact their patients’ quality of life and their patients’ life expectancy, respectively. Only 14% of the societies expected that drug accessibility will improve in the next 5 years, while 45% anticipated no change and 28% expected the situation to worsen. Finally, 78% of the societies considered that national policy changes could improve drug accessibility, while two-thirds believed that European policy changes could improve antiarrhythmic drug accessibility.

## Discussion

### Severe anti-arrhythmic drug accessibility problems within and outside the European Union

With these successive surveys, we established contemporary insights into accessibility issues surrounding antiarrhythmic drugs across the EU and beyond. Despite our reliance on questionnaires with a potential for bias (see Limitations), most of these drugs are thought of as standard of care and are recommended in international guidelines and consensus statements.^20–23^ The results show that accessibility varies over time, between countries, and even within countries. Nevertheless, it is evident that clinicians frequently encounter difficulties in obtaining antiarrhythmic drugs for their patients, creating important barriers to optimal arrhythmia care. These findings reflect the fragile supply chains and access pathways for antiarrhythmic drugs, highlighting systemic vulnerabilities that extend across national and institutional boundaries. Importantly, all these drugs are off-patent and are inexpensive to produce. Mexiletine represents a notable exception in commercial terms, having been expropriated and repositioned within the rare disease market with a price multiplied by hundreds despite low production costs.^11,13^ Another important issue is the formulations for children, especially for AAD without appropriate options for substitution (e.g. mexiletine solution, see Supplementary material online, *Figure S2*). Especially for drugs that have low use volumes (such as mexiletine solution and oral formulations or ajmaline i.v.), the commercial revenue might be considered too small for companies to market such drugs without appropriate consideration of the need for these drugs. Such challenges with treatment are extremely disturbing for physicians, patients, and their families.

### Impact on patients’ arrhythmia treatment

The overall findings are highly concerning, even acknowledging the limitations inherent to survey-based research and the restricted ability to draw robust country-level conclusions from small sample sizes. Antiarrhythmic drugs continue to have specific roles in contemporary clinical practice, and for several drugs, there is no or only inadequate substitution.^10,11,24^ Limited accessibility may therefore force clinicians to make considerable efforts to reach a diagnosis (e.g. ajmaline i.v.) or obtain treatment, delay treatment, or adapt treatment decisions according to local constraints rather than clinical preference (e.g. propranolol or nadolol in Long QT syndrome, or using amiodarone over other AAD with less toxicity issues).^25^ In that context, the perception of most national societies that these accessibility issues negatively impact patients’ quality of life and, in some cases, life expectancy is noteworthy, albeit that neither the impact on quality of life nor life expectancy could be directly measured.

It is also important to distinguish between reported or perceived accessibility and formal availability as recorded in regulatory databases, such as those maintained by the European Medicines Agency,^26^ or on national levels.^27^ Patient access involves far more than the mere existence of marketing authorization or product availability; it also depends on, e.g. reimbursement decisions, import options, distribution capacity, and institutional resources. This survey primarily captures the perceptions and experiences of respondents rather than objective accessibility data. This is both a strength and a limitation: it reflects the reality faced by prescribers, but it is also susceptible to variation in local pathways, institutional support, and individual perception. Simultaneously, physicians will often adjust their local management strategies to the availability of specific drugs,^28^ which may in turn influence the perceived accessibility problems and, in some cases, compromise optimal arrhythmia management.

### Accessibility and availability problems with anti-arrhythmic drugs

Another important factor is whether a medicine is registered. Medicines can be marketed via a national marketing authorization or, in Europe, via a centralized EU procedure at the European Medicines Agency. Although a central EU registration ensures that a product is approved for use in all member states, actual access for patients depends on national reimbursement decisions, which remain the exclusive competence of each individual EU country. In the case of a national marketing authorization in another country, it is sometimes possible to import a medicine or use a local pharmacy preparation. The routes to availability are, however, not always clear; they can take a lot of time, and reimbursement is often not properly arranged. Notably, in the case of production chain or distribution issues, the absence of national marketing authorization holders or reliance on only one marketing authorization holder can have direct consequences for drug accessibility and thus makes countries more vulnerable to accessibility issues.^29^

Most respondents worked in academic centres with extensive expertise in arrhythmia management and relatively strong institutional resources. Nevertheless, even in these settings, prescribers remain highly dependent on pharmacists with sufficient experience, time, and resources to obtain specific drugs, as well as on local frameworks and budgets that either facilitate or hinder access. These include national legislation, import infrastructure, and local capacity for pharmacy preparations. In addition, the surveys were conducted over different time periods, and drug accessibility is known to fluctuate between and within countries. This is also evident from the differing results of the initial and second surveys, which demonstrate that local accessibility can vary over time. Despite this variability, accessibility problems remained substantial in both survey rounds, highlighting the persistence of these challenges.

### Potential causes of inequalities in access to antiarrhythmic drugs

Another finding from the survey is the inferred association between national income levels and the number of market authorization holders, with the assumption that a higher number of marketing authorizations for a given product generally corresponds to better availability.^30^ Notwithstanding the fact that we could not perform formal association analyses between income levels and number of market authorization holders, these two factors are likely interrelated; higher-income countries typically offer greater profit potential for market authorization holders and therefore tend to maintain a broader portfolio of registered products. Consequently, globally, a highly heterogeneous landscape persists in the range of antiarrhythmic drugs accessible on national markets. This may also help explain why accessibility problems persist even for older drugs that are inexpensive to produce. The issue is not simply the cost of manufacturing but also the fragility of the surrounding ecosystem: limited commercial incentives, dependence on a small number of suppliers, administrative barriers to importation or reimbursement, and lack of redundancy within supply chains. In addition, wealthier countries are often able to obtain medicines from other markets through parallel import mechanisms, temporarily alleviating shortages within their own borders. However, this same process can exacerbate accessibility problems in lower-income countries, where supply chains are more vulnerable, price margins are smaller, and the ability to pay or reimbursement is less, as was shown earlier for other (non-AAD) drugs.^31,32^ Thus, parallel import may unintentionally intensify existing disparities in access to medicines inside and outside the EU.

### Fragile pharmaceutical supply chains and strategies

These findings on antiarrhythmic drugs mirror broader concerns raised by policymakers and researchers, who have repeatedly highlighted fragile pharmaceutical supply chains and (Europe’s) heavy reliance on Asian manufacturers as key drivers of drug availability problems.^33–36^ However, recent analyses of upstream pharmaceutical supply chains indicate that vulnerabilities cannot be completely resolved in Europe by increasing active pharmaceutical ingredient (API) production within the EU. While API manufacturing is largely concentrated in Asia, a substantial share of finished product manufacturing already occurs in the EU.^37^ Still, security of supply remains precarious due to limited diversification, over-reliance on single manufacturers, and the absence of robust dual-sourcing strategies. Importantly, vulnerabilities are not confined to medicines officially labelled as ‘critical’ at the EU level,^38^ as similar concerns may apply to antiarrhythmic drugs not classified as ‘critical’. Consequently, the scope of initiatives such as the EU’s Critical Medicines Act should be broadened. Beyond a narrow focus on a predefined list of ‘critical medicines’, policy should also safeguard drugs with high clinical impact and societal relevance, including antiarrhythmic drugs.

### Equitable access requires coordinated action

Respondents widely agreed that both national and (for the EU) EU-wide measures are required to address the persistent and multifactorial barriers to patient access. To design effective interventions, future research should identify and quantify the root causes of these accessibility problems.

Taken together, the present surveys suggest that antiarrhythmic drug accessibility problems are persistent, multifactorial, and clinically relevant. They also indicate that action is required both on national and EU levels. Future work should aim to move beyond clinician-reported experience and integrate regulatory, economic, reimbursement, and manufacturing data to identify the main drivers of poor accessibility more precisely. Such work will be essential to guide effective interventions and to support a more resilient and equitable framework for access to antiarrhythmic therapy. This is in line with the emerging perspective of supporting a patient-centred, value-based health care, with the aim to reduce unwarranted variations in access and inequities that constitute a barrier to appropriate quality of health.^39^ Ultimately, securing reliable and equitable access to these drugs for all patients will require coordinated action from clinicians, professional societies, policymakers, manufacturers, and institutions.

## Limitations

There are certainly limitations to this work, as also already mentioned regarding the limited number of responses from individual countries, the variable number and demographics of respondents per country (troubling actual nationwide accessibility problems), and measurements of perception rather than actual outcome data. In addition, in adherence to the General Data Protection Regulation and like previous ESC-EHRA surveys, this was an anonymous survey. Although a general evaluation of the survey results did not reveal duplications or fake entries, we cannot confirm with certainty that this has not happened. Thirdly, bias in response vs. non-response for any reason will also be relevant to this survey.

## Conclusions

Physicians worldwide frequently encounter problems in accessing guideline-recommended antiarrhythmic drugs for their patients. Reported accessibility varied markedly across countries, within countries, and over time, highlighting the fragility of current access pathways. These findings suggest that antiarrhythmic drug accessibility problems are persistent, multifactorial, and clinically relevant, with potential consequences for quality of care and patient outcomes. Addressing these challenges will require coordinated action from clinicians, professional societies, policymakers, manufacturers, and institutions to ensure timely and equitable access to essential antiarrhythmic therapy.

## Supplementary Material

euag220_Supplementary_Data

## Data Availability

The data underlying this article will be shared on reasonable request to the corresponding author.
