# Supplementary material for: Essential antiarrhythmic drug accessibility worldwide: a multi-survey study and comprehensive evaluation of access and supply challenges
Source: Europace. 2026 Sep 2;28(9):euag220. doi: 10.1093/europace/euag220 (PMC13554807; doi:10.1093/europace/euag220)
Supplement: Supplementary file 1 [file euag220_supplementary_data.zip › EUPC-D-26-00937_R1 clean.pdf]

Manuscript (incl.refs, Acknowledgments, Funding, Ethics, etc follow guidelines)

Click here to access/download;Manuscript (incl.refs, Acknowledgments, Funding, Ethics, etc follow

Essential antiarrhythmic drug accessibility worldwide: A multi-survey study and comprehensive evaluation of access and supply challenges.

Pieter G. Postema, MD, PhD[1,2,3]

Niels D. Reijnhout, MSc[4*]

Federico Ballacci, MD[5*]

Evert A. Manders, MSc[4,6*]

Wiert F. Hoeksema, MD[1*]

Mauro Toniolo, MD[2,3,5]

Jacob Tfelt-Hansen, MD, PhD[3,7]

Diego Penela, MD, PhD[8]

Piotr Futyma, MD[9]

Deirdre Lane, PhD[2,10,11]

Ruben Casado-Arroyo, MD, PhD[2,12]

Georgia Sarquella-Brugada, MD, PhD[2,3,13,14]

Nico A. Blom, MD, PhD[3, 14,15]

Arthur A.M. Wilde, MD, PhD[1,3]

Carla E.M. Hollak, MD, PhD[4,6]

Elena Arbelo, MD, PhD[2,3,8,17,18]

* Contributed equally

Affiliations

1. Department of Clinical and Experimental Cardiology, Heart Center, Amsterdam UMC, University of Amsterdam, Cardiovascular Sciences, Amsterdam, The Netherlands
2. European Heart Rhythm Association Advocacy, Quality Improvement and Health Economics Committee (EHRA-AQIHEC)
3. European Reference Network (ERN) GUARD-Heart for rare and complex diseases of the heart
4. Medicine for Society Platform at Amsterdam University Medical Centers, University of Amsterdam, Amsterdam, The Netherlands
5. Cardiology Division, University Hospital "S. Maria della Misericordia", Udine, Italy
6. Department of Endocrinology and Metabolism, Amsterdam University Medical Centers, University of Amsterdam, Amsterdam, The Netherlands
7. Department of Cardiology, Heart Centre, Copenhagen University Hospital, Rigshospitalet, and Department of Forensic Medicine, Faculty of Medical Sciences, University of Copenhagen, Copenhagen, Denmark.
8. Arrhythmia Section, Cardiology Department, Hospital Clinic, Universitat de Barcelona, Spain
9. St. Joseph's Heart Rhythm Center, University of Rzeszów, Rzeszów, Poland
10. Department of Cardiovascular and Metabolic Medicine, Institute of Life Course and Medical Sciences, University of Liverpool, Liverpool, UK
11. Liverpool Centre for Cardiovascular Sciences, University of Liverpool, Liverpool John Moores University and Liverpool Heart and Chest Hospital, Liverpool, UK

12. Department of Cardiology, Erasme University Hospital, Université Libre de Bruxelles, Brussels, Belgium.
13. Arrhythmias, Inherited Cardiac Diseases and Sudden Death Unit, Hospital Sant Joan de Déu, Esplugues de Llobregat, Barcelona, Spain
14. Association for European Paediatric and Congenital Cardiology (AEPC)
15. Department of Pediatric Cardiology, Amsterdam UMC, University of Amsterdam, Emma Children's Hospital, Amsterdam, The Netherlands
16. Association for European Paediatric and Congenital Cardiology (AEPC)
17. Institut d'Investigacio August Pi i Sunyer (IDIBAPS), Barcelona, Spain
18. Centro de Investigaci on Biome´dica en Red de Enfermedades Cardiovasculares (CIBERCV), Madrid, Spain

Corresponding author: Dr. Pieter G. Postema, Heart Center, Department of Clinical and Experimental Cardiology, Amsterdam UMC, University of Amsterdam, Cardiovascular Sciences, Meibergdreef 9, Amsterdam 1105 AZ, the Netherlands. Email: P.G.Postema@amsterdamumc.nl . Tel: +31-20-5663072.

Funding / Conflicts of interest

PGP is funded by a grant of the *Dutch Heart Foundation (03-003-2021-T061)*
Other authors report no conflicts of interest.

Abstract

Background and aims. Antiarrhythmic drugs (AADs) are essential for preventing symptoms, morbidity, and mortality associated with cardiac arrhythmias. However, concerns are increasing about their accessibility. We aimed to assess insights into current access to AADs.

Methods. Online surveys were distributed to members of the European Heart Rhythm Association (EHRA), the Association for European Paediatric and Congenital Cardiology, the European Reference Network GUARD-Heart, and national heart rhythm societies.

Results. Responses were obtained from 558 respondents across 80 countries. Among countries included in the main analysis (≥5 respondents; 16 countries within and 7 outside the European Union, n=298 respondents), only 16–59% of surveyed AADs were considered readily accessible. Accessibility varied substantially by drug: oral flecainide and propranolol were available in 88% of countries, whereas mexiletine solution was available in only 10%. Marked differences in accessibility were reported both between and within countries, with availability also changing over time. Higher-income countries generally reported better access and a greater number of market authorization holders than lower-income countries. National societies' perception was that limited access negatively affected patients' quality of life (72%) and life expectancy (35%), while 73% expected these challenges to persist or worsen in the coming years.

Conclusion. Physicians worldwide frequently encounter problems accessing guideline recommended antiarrhythmic drugs for their patients, which putatively impacts on their patients' quality of life and life expectancy. This highlights systemic vulnerabilities that extend across national and institutional boundaries, urging close

collaboration among clinicians, policymakers, manufacturers, and institutions to guarantee timely and equitable treatment for all patients.

What's new.

1. Multiple antiarrhythmic drugs, recommended in international guidelines, are not readily accessible in any country worldwide, with country-level coverage ranging from 16% to 59% of surveyed drugs.
2. Striking differences exist between and within countries regarding antiarrhythmic drug accessibility.
3. Lower reported antiarrhythmics accessibility appeared to coincide with fewer market authorisation holders and lower national income, albeit that no formal association analyses could be performed on these data.
4. National societies perceived these accessibility problems to affect patients' quality of life and, in some cases, life expectancy, highlighting persistent systemic vulnerabilities in arrhythmia care.

Key words.

Antiarrhythmic drugs, drug accessibility, medicines shortages, arrhythmia care, survey

Graphical abstract

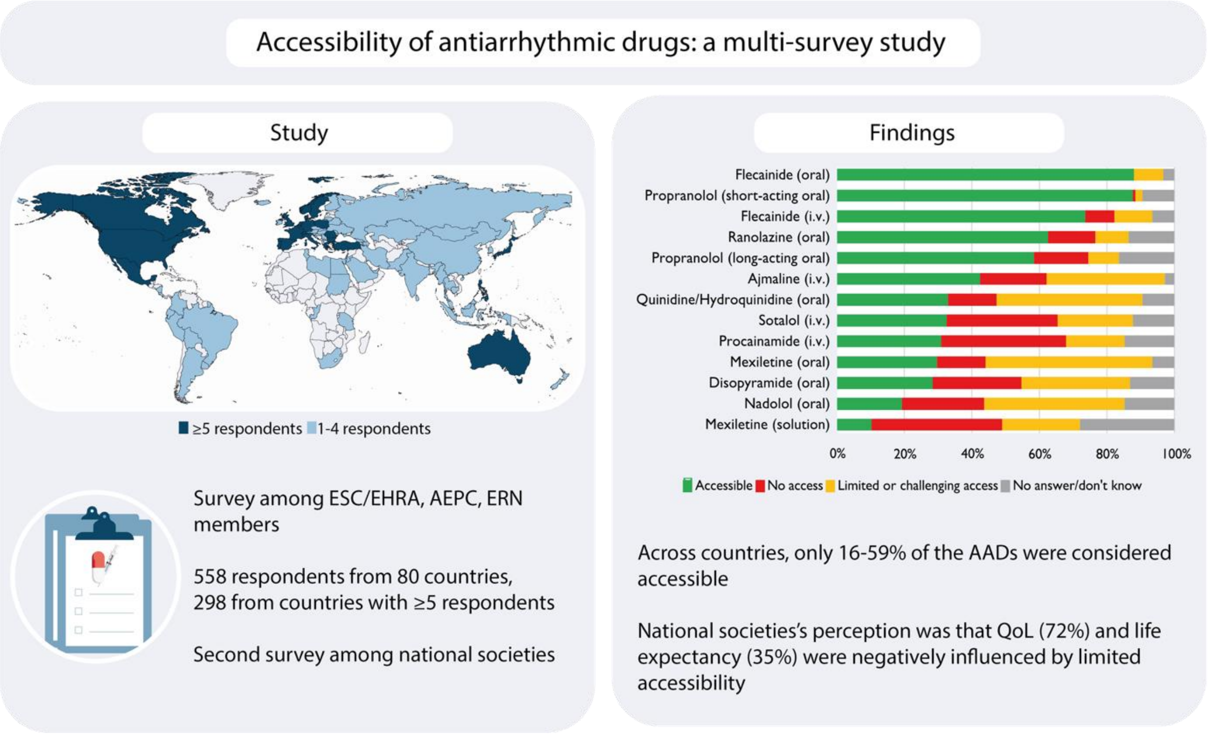

Introduction

Antiarrhythmic drugs remain essential for the prevention and treatment of cardiac arrhythmias, improving symptoms and, in selected cases, reducing morbidity and mortality. The choice for a certain antiarrhythmic drug is dependent on the specific arrhythmia, the underlying mechanisms resulting in the arrhythmia, it's burden and prognosis, comorbidities (such as structural heart disease, heart-, kidney- and liver function), tolerability, and, equally important, accessibility. The development of nearly all antiarrhythmic drugs dates to the 1960s. 1970s, and 1990s (Figure 1). Over the last 30 years, there has been sparse development in this field, later examples being ivabradine (1994),[1] dronedarone (1995),[2] and vernakalant (2007).[3] The number of patients with an arrhythmia has increased exponentially in the past decades, causing sharp initial increases in antiarrhythmic drug use but with successive declines over time in the past years in several regions, putatively due to the limited accessibility of antiarrhythmic drugs.[4–7] Thus, there is a pressing need to improve access to these drugs on a global scale. Antiarrhythmic drugs often have limited options for substitution by other drugs, increasing the implications of accessibility problems. If substitution is available, strict guidance and monitoring are required for successful application.

Accessibility has several dimensions, including regulatory approval, commercial availability, reimbursement, local formulary restrictions, affordability, and (temporary) shortages, which may differ considerably between and even within countries. Availability and affordability are the most important.[8] For antiarrhythmic drugs, these dimensions are particularly important because limitations in access may affect both acute and long-term management across a broad range of arrhythmias.

Well-recognised accessibility examples include quinidine and mexiletine to treat malignant arrhythmia, illustrating how access problems arise when clinically important drugs become commercially fragile, difficult to source, or poorly supported by existing regulatory and reimbursement pathways.[9–11] From these and other examples, multiple causes of these accessibility issues emerged, two major causes being an increased dependency on fewer supply chains and withdrawal of products from the market due to commercial reasons.[12,11,13] In parallel, concerns about pro-arrhythmic risk and the substantial cost and complexity of drug development may have discouraged further innovation in antiarrhythmic therapy.[14–17]

To evaluate accessibility issues and to investigate the future perspective regarding antiarrhythmic drugs in Europe and beyond, the Advocacy, Quality Improvement and Health Economics Committee (AQIHEC) of the European Heart Rhythm Association (EHRA) and the Association for European Paediatric and Congenital Cardiology (AEPC), together with the European Society of Cardiology (ESC) and the European Reference Network (ERN) GUARD-Heart for rare and complex diseases of the heart, launched a survey amongst their members. In this survey we aimed to establish a current view of the extent of problems that exist for physicians to acquire antiarrhythmic drugs for their patients and to explore factors influencing the accessibility. These antiarrhythmic drugs cover both orphan drugs used to treat selected patients at risk for sudden death by specific malignant arrhythmia syndromes (who can be totally dependent on a single active ingredient, e.g. quinidine), as well as antiarrhythmic drugs used for very common but more benign arrhythmias such as atrial fibrillation.

Methods

Study design and survey development

This study was based on successive international online surveys designed to assess physician-reported accessibility of antiarrhythmic drugs, reflecting whether respondents considered a given drug obtainable for patient care within their practice setting, and to explore variation in access within and across countries. The survey instrument was developed iteratively with input from the EHRA-AQIHEC, AEPC, ESC, and the ERN GUARD-Heart. After evaluation and permission by the appropriate ESC-EHRA, AEPC and ERN-Guard Heart bodies, similar to previous ESC-EHRA surveys,[18] the survey was designed online using SurveyMonkey with support of the ESC-EHRA staff (see the Supplement). Notably, to adhere to the General Data Protection Regulation, the survey was designed to only capture anonymous data. A link to the survey was distributed through email to EHRA, AEPC and ERN-Guard Heart members and peer networks.

For the main analysis, the writing group selected antiarrhythmic drugs considered clinically relevant and potentially vulnerable to accessibility problems. The main analyses were subsequently limited to the following drugs: mexiletine (oral and suspension), nadolol (oral), propranolol (oral short acting and long acting), ajmaline (i.v.), procainamide (i.v.), disopyramide (oral), sotalol (i.v.), flecainide (oral and i.v.), and ranolazine (oral). Data regarding the other drugs are provided in the Supplementary materials.

Survey analyses

The respondents were characterized based on country, primary workplace, profession, career stage, patient category (adult/pediatric) and experience in

treatment decision making regarding different arrhythmias. Respondents who did not answer any of the questions regarding accessibility of specific drugs were excluded. For the analysis we included only countries with five or more respondents. Countries with less than 5 respondents were only categorized but not further investigated in detail to pertain a reasonable minimal standard for adequate data collection. While EHRA-member countries also cover parts of Northern Africa and Western Asia, and individual EHRA-members can be located all over the world, there is significant influence of the European Union (EU) on national legislation and regulation on drug accessibility. Therefore, we evaluated responses from countries within the EU (including Norway, Switzerland and the United Kingdom, who comply largely to EU regulation) separately from responses from countries outside the EU (e.g. Australia, Canada, Japan, Mexico, Philippines, Turkey and the United States of America).

In an extended accessibility analysis limited to EU countries plus the United Kingdom, we explored the number of marketing authorization holders for the active substances of these drugs per country as a potential factor influencing accessibility. Data were obtained from the EU Article 57 database, the repository of all medicines authorized in the European Economic Area, with additional investigations for the United Kingdom.

Follow-up consultation with national societies

To validate and expand on these results, we sent out a summary of the national results of the initial survey to the national (heart rhythm) societies asking for their reflections. This was done for the EHRA countries with any number of respondents and for the countries outside EHRA with ≥5 respondents. With this additional round of characterization, we aimed to strengthen the respondent's perceived availability

issues, to possibly gain more insights in the national regulative affairs that may hamper availability, and to collect a reflection in a different time frame (about a year later) to see whether availability issues change over time. This round of evaluations was submitted in the first half of 2025 and 2026.

Results

Respondent characteristics and survey coverage

The survey was distributed at the end of 2023 through mailings, social media and peer networks. It attracted 558 respondents, mainly from Europe, but also from the Americas, Africa, Asia and Oceania. Most respondents were male (66%), between 40-49 years of age (30%), working in academia (64%), in cardiac electrophysiology (59%), treating adults (75%), children (8%) or both (17%), and making daily or weekly treatment decisions for atrial fibrillation (80%), ventricular tachycardia (63%) and rare or inheritable arrhythmia syndromes (32%).

In total, we received responses from 43 out of 55 EHRA member countries (78%), and from 37 countries outside EHRA member countries. An overview of the accessibility of all antiarrhythmic drugs reported in this survey for countries with ≥5 respondents (16 countries within and 7 outside the EU, n=298 respondents) is presented in Figure 2, separated by countries within the EU (A) and outside the EU (B), while Figure 3A and 3B illustrate the accessibility of individual products from the main analysis within these countries.

Although the response rate to this survey ranks among the highest for ESC surveys, the wide range of respondents and divergent results meant that country-level analyses, even within the EU, were limited by small sample sizes. Nevertheless, several observations could be made.

Accessibility across countries and drugs

The accessibility of antiarrhythmic drugs varied substantially across countries inside and outside the EU. For the EU, overall, Romania showed the lowest accessibility, with only 16% of the assessed products being readily accessible, whereas the United Kingdom demonstrated the highest accessibility, with 59% of products being reported as readily accessible (Figure 2A). Accessibility tended to be higher in countries with greater national income compared to those with lower income levels within the EU (Figure 2). albeit that no formal association analyses could be performed on these data. Sweden appeared to be an outlier in this regard, with respondents declaring relatively limited accessibility despite its high national income.[19]

Outside the EU, overall, Turkey showed the lowest reported availability, with only 20% of the assessed products considered readily accessible, whereas the USA demonstrated the highest accessibility, with 59% of products being reported as readily accessible (Figure 2B).

Regarding individual products, oral flecainide and propranolol were the most widely available both inside and outside the EU, reported as readily accessible in 88% of countries. In contrast, mexiletine (solution) was readily accessible in only about 10-12% of countries inside and outside the EU, making it one of the least accessible antiarrhythmic drugs together with ajmaline (3%) and i.v. flecainide (19%) outside the EU (Figure 3A and 3B). More data on the other surveyed AAD inside and outside the EU are available from the Supplemental Figures 1-3.

Variability within countries and over time

Some antiarrhythmic drugs show accessibility issues across different countries – suggesting that some antiarrhythmic drugs have broad cross-national accessibility issues. Examples are mexiletine, nadolol and procainamide. However, there is high variability in accessibility; while certain antiarrhythmic drugs are accessible in one country, they are not accessible in another country, and vice versa. An example of this pattern is ranolazine (see Supplemental Figure 2). In some countries, however, accessibility issues for certain antiarrhythmic drugs are (very) differently perceived, suggesting that some physicians have access, but others do not or experience difficulties obtaining the drugs. Examples hereof are disopyramide, sotalol and procainamide in multiple countries.

Exploratory analysis of market authorisation holders

After an extended accessibility analysis of these results (limited to EU countries plus the United Kingdom), the following additional observations could be made:

1. Reported accessibility appeared to be greater in countries with higher numbers of registered products for the relevant active ingredient (i.e., a certain antiarrhythmic drug/active ingredient may be registered by 20 companies in one country but only one in another country). For example, France, Germany, and the United Kingdom have relatively high numbers of registered products (≥35) and reported fewer accessibility issues.
2. There is a marked difference in the number of registered products across different drugs. Propranolol, flecainide, sotalol and ranolazine have over 50 market authorizations in the EU member states, while all others have 11 or fewer.

3. If antiarrhythmic drugs have significant accessibility issues, it appears that the number of registered drugs is low. Illustrations of these differences are depicted in Figures 4 and 5.

Additional insights in the survey results, including from the countries with <5 respondents and outside the EU, are available in the figures in the supplement. Also, barriers and solutions as perceived by the respondents, as well as data on coverage of the costs associated with the surveyed drugs can be found in the supplements (Supplemental Figures 1-5).

Perception by national (heart rhythm) societies

From 41 of the 43 EHRA member countries with respondents to the initial survey (Russia and Belarus are currently suspended from the ESC and were thus not contacted), we finally received responses from 34 (83%) national (heart rhythm) societies to the summaries of their country results. Of the 6 countries with ≥5 respondents outside EHRA members countries, we received 5 responses (86%). On average, 71% agreed or strongly agreed with the outcomes of the initial survey on a per-drug basis, however, 22% disagreed or strongly disagreed. Remarks provided to these disagreements mirrored the general observations mentioned above for the initial survey: namely the divergence of accessibility. While some antiarrhythmic drugs were considered easily accessible by the individual respondents, a year later they were hard to acquire in the views of the societies, and vice versa (for example, nadolol, mexiletine and ranolazine). In addition, even antiarrhythmic drugs previously regarded as reasonable accessible (and not included in the main survey results, such as oral amiodarone and oral sotalol), were mentioned to have problems with

accessibility (see Supplemental Figures 3A and 3B). Arguably, this may in part be due to different time frames in which these surveys were gathered, making them prone to disruption or improvement in accessibility between the surveys. In addition, 72% and 35% of the societies feel that accessibility issues negatively impact on their patients' quality of life and their patients' life expectancy, respectively. Only 14% of the societies expected that drug accessibility will improve in the next 5 years, while 45% anticipated no change and 28% expected the situation to worsen. Finally, 78% of the societies considered that national policy changes could improve drug accessibility, while two-thirds believed that European policy changes could improve antiarrhythmic drug accessibility.

Discussion

Severe anti-arrhythmic drugs accessibility problems within and outside the European Union

With these successive surveys, we established contemporary insights into accessibility issues surrounding antiarrhythmic drugs across the EU and beyond. Despite our reliance on questionnaires with a potential for bias (see Limitations), most of these drugs are thought of as standard of care and are recommended in international guidelines and consensus statements.[20–23] The results show that accessibility varies over time, between countries, and even within countries. Nevertheless, it is evident that clinicians frequently encounter difficulties in obtaining antiarrhythmic drugs for their patients, creating important barriers to optimal arrhythmia care. These findings reflect the fragile supply chains and access pathways for antiarrhythmic drugs, highlighting systemic vulnerabilities that extend across national and institutional boundaries. Importantly, all these drugs are off-

patent and are inexpensive to produce. Mexiletine represents a notable exception in commercial terms, having been expropriated and repositioned within the rare-disease market with a price multiplied by hundreds despite low production costs.[11,13] Another important issue are the formulations for children, especially for AAD without appropriate options for substitution (e.g. mexiletine solution, see Supplemental Figure 2). Especially for drugs that have low use-volumes (such as mexiletine solution and oral formulations, or ajmaline i.v.) the commercial revenue might be considered too small for companies to market such drugs, without appropriate considerations of the need for these drugs. Such challenges with treatment are extremely disturbing for physicians, patients and their families.

Impact on patients' arrhythmia treatment

The overall findings are highly concerning, even acknowledging the limitations inherent to survey-based research and the restricted ability to draw robust country-level conclusions from small sample sizes. Antiarrhythmic drugs continue to have specific roles in contemporary clinical practice, and for several drugs there is no or only inadequate substitution.[10,11,24] Limited accessibility may therefore force clinicians to make considerable efforts to reach a diagnosis (e.g. ajmaline i.v.) or obtain treatment, and delay treatment, or adapt treatment decisions according to local constraints rather than clinical preference (e.g. propranolol or nadolol in Long QT syndrome, or using amiodarone over other AAD with less toxicity issues).[25] In that context, the perception of most national societies that these accessibility issues negatively impact on patients' quality of life, and in some cases on life expectancy, is noteworthy. Albeit that the impact on quality of life nor life expectancy could not be directly measured.

It is also important to distinguish between reported or perceived accessibility and formal availability as recorded in regulatory databases such as those maintained by the European Medicines Agency,[26] or on national levels.[27] Patient access involves far more than the mere existence of marketing authorization or product availability; it also depends on, e.g., reimbursement decisions, import options, distribution capacity, and institutional resources. This survey primarily captures the perceptions and experiences of respondents rather than objective accessibility data. This is both a strength and a limitation: it reflects the reality faced by prescribers, but it is also susceptible to variation in local pathways, institutional support, and individual perception. Simultaneously, physicians will often adjust their local management strategies to the availability of specific drugs,[28] which may in turn influence the perceived accessibility problems and, in some cases, compromise optimal arrhythmia management.

Accessibility and availability problems with anti-arrhythmic drugs

Another important factor is whether a medicine is registered. Medicines can be marketed via a national marketing authorization or, in Europe, via a centralized EU procedure at the European Medicines Agency. Although a central EU registration ensures that a product is approved for use in all member states, actual access for patients depends on national reimbursement decisions, which remain the exclusive competence of each individual EU country. In the case of a national marketing authorization in another country, it is sometimes possible to import a medicine or use a local pharmacy preparation. The routes to availability are, however, not always clear, they can take a lot of time and reimbursement is often not properly arranged. Notably, in the case of production chain or distribution issues, the absence of national

marketing authorization holders or reliance on only one marketing authorization holder, can have direct consequences for drug accessibility and thus makes countries more vulnerable to accessibility issues.[29]

Most respondents worked in academic centres with extensive expertise in arrhythmia management and relatively strong institutional resources. Nevertheless, even in these settings, prescribers remain highly dependent on pharmacists with sufficient experience, time, and resources to obtain specific drugs, as well as on local frameworks and budgets that either facilitate or hinder access. These include national legislation, import infrastructure, and local capacity for pharmacy preparations. In addition, the surveys were conducted over different time periods, and drug accessibility is known to fluctuate between and within countries. This is also evident from the differing results of the initial and second surveys, which demonstrate that local accessibility can vary over time. Despite this variability, accessibility problems remained substantial in both survey rounds, highlighting the persistence of these challenges.

Potential causes of inequalities in access to antiarrhythmic drugs

Another finding from the survey is the inferred association between national income levels and the number of market authorization holders, with the assumption that a higher number of marketing authorizations for a given product generally corresponds to better availability.[30] Notwithstanding the fact that we could not perform formal association analyses between income levels and number of marker authorization holders, these two factors are likely interrelated; higher-income countries typically offer greater profit potential for market authorization holders and therefore tend to maintain a broader portfolio of registered products. Consequently, globally a highly

heterogeneous landscape persists in the range of antiarrhythmic drugs accessible on national markets. This may also help explain why accessibility problems persist even for older drugs that are inexpensive to produce. The issue is not simply the cost of manufacturing, but also the fragility of the surrounding ecosystem: limited commercial incentives, dependence on a small number of suppliers, administrative barriers to importation or reimbursement, and lack of redundancy within supply chains. In addition, wealthier countries are often able to obtain medicines from other markets through parallel import mechanisms, temporarily alleviating shortages within their own borders. However, this same process can exacerbate accessibility problems in lower-income countries, where supply chains are more vulnerable, price margins are smaller and ability to pay or reimbursement is less, as was shown earlier for other (non-AAD) drugs.[31,32] Thus, parallel import may unintentionally intensify existing disparities in access to medicines inside and outside the EU.

Fragile pharmaceutical supply chains and strategies

These findings on antiarrhythmic drugs mirror broader concerns raised by policymakers and researchers, who have repeatedly highlighted fragile pharmaceutical supply chains and (Europe's) heavy reliance on Asian manufacturers, as key drivers of drug availability problems.[33–36] However, recent analyses of upstream pharmaceutical supply chains indicate that vulnerabilities cannot be completely resolved in Europe by increasing active pharmaceutical ingredient (API) production within the EU. While API manufacturing is largely concentrated in Asia, a substantial share of finished product manufacturing already occurs in the EU.[37] Still, security of supply remains precarious due to limited diversification, over-reliance on single manufacturers, and the absence of robust dual-sourcing strategies.

Importantly, vulnerabilities are not confined to medicines officially labelled as “critical” at the EU level,[38] as similar concerns may apply to antiarrhythmic drugs not classified as “critical”. Consequently, the scope of initiatives such as the EU’s Critical Medicines Act should be broadened. Beyond a narrow focus on a predefined list of “critical medicines,” policy should also safeguard drugs with high clinical impact and societal relevance, including antiarrhythmic drugs.

Equitable access requires coordinated action

Respondents widely agreed that both national and (for the EU) EU-wide measures are required to address the persistent and multifactorial barriers to patient access. To design effective interventions, future research should identify and quantify the root causes of these accessibility problems.

Taken together, the present surveys suggest that antiarrhythmic drug accessibility problems are persistent, multifactorial, and clinically relevant. They also indicate that action is required both on national and EU levels. Future work should aim to move beyond clinician-reported experience and integrate regulatory, economic, reimbursement, and manufacturing data to identify the main drivers of poor accessibility more precisely. Such work will be essential to guide effective interventions and to support a more resilient and equitable framework for access to antiarrhythmic therapy. This is in line with the emerging perspective of supporting a patient-centered value based health care, with the aim to reduce unwarranted variations in access, and inequities that constitute a barrier to appropriate quality of health.[39] Ultimately, securing reliable and equitable access to these drugs for all patients will require coordinated action from clinicians, professional societies, policymakers, manufacturers, and institutions.

Limitations

There are certainly limitations to this work, as also already mentioned regarding the limited number of responses from individual countries, the variable number and demographics of respondents per country (troubling actual nationwide accessibility problems), and measurements of perception rather than actual outcome data. In addition, in adherence to General Data Protection Regulation and like previous ESC-EHRA surveys, this was an anonymous survey. Although a general evaluation of the survey results did not reveal duplications or fake entries, we cannot confirm with certainty that this has not happened. Thirdly, bias in response versus non-response for any reason will also be relevant to this survey.

Conclusions

Physicians worldwide frequently encounter problems in accessing guideline recommended antiarrhythmic drugs for their patients. Reported accessibility varied markedly across countries, within countries, and over time, highlighting the fragility of current access pathways. These findings suggest that antiarrhythmic drug accessibility problems are persistent, multifactorial and clinically relevant, with potential consequences for quality of care and patient outcomes. Addressing these challenges will require coordinated action from clinicians, professional societies, policymakers, manufacturers, and institutions, to ensure timely and equitable access to essential antiarrhythmic therapy.

Acknowledgements

We would like to express our sincere gratitude to all colleagues and all national societies who have participated in the surveys. We are particularly grateful to Lauren Tapp and Vanessa Meyen and their colleagues from the EHRA bureau for organizing and supporting the survey distributions.

References

1. Thollon C, Cambarrat C, Vian J, Prost JF, Peglion JL, Vilaine JP. Electrophysiological effects of S 16257, a novel sino-atrial node modulator, on rabbit and guinea-pig cardiac preparations: comparison with UL-FS 49. *Br J Pharmacol*. 1994;112:37–42.
2. Manning AS, Bruyninckx C, Ramboux J, Chatelain P. SR 33589, a new amiodarone-like agent: effect on ischemia- and reperfusion-induced arrhythmias in anesthetized rats. *J Cardiovasc Pharmacol*. 1995;26:453–461.
3. Vernakalant: RSD 1235, RSD-1235, RSD1235. *Drugs R D*. 2007;8:259–265.
4. Piccinni C, Raschi E, Poluzzi E, Puccini A, Cars T, Wettermark B, Diemberger I, Boriani G, De Ponti F. Trends in antiarrhythmic drug use after marketing authorization of dronedarone: comparison between Emilia Romagna (Italy) and Sweden. *Eur J Clin Pharmacol*. 2013;69:715–720.
5. Dev S, Peterson PN, Wang Y, Curtis JP, Varosy PD, Masoudi FA. Prevalence, correlates, and temporal trends in antiarrhythmic drug use at discharge after implantable cardioverter defibrillator placement (from the National Cardiovascular Data Registry [NCDR]). *Am J Cardiol*. 2014;113:314–320.
6. Markman TM, Geng Z, Epstein AE, Nazarian S, Deo R, Marchlinski FE, Groeneveld PW, Frankel DS. Trends in Antiarrhythmic Drug Use Among Patients in the United States Between 2004 and 2016. *Circulation*. 2020;141:937–939.
7. Poulsen CB, Damkjær M, Løfgren B, Schmidt M. Trends in Antiarrhythmic Drug Use in Denmark Over 19 Years. *Am J Cardiol*. 2020;125:562–569.
8. Lotfizadeh A, Palafox B, Takallou A, Balabanova D, McKee M, Murphy A. Factors associated with the availability and affordability of essential cardiovascular disease medicines in low- and middle-income countries: A systematic review. *PLOS Glob Public Health*. 2022;2:e0000072.
9. Wilde AA, Langendijk PN. Antiarrhythmic drugs, patients, and the pharmaceutical industry: value for patients, physicians, pharmacists or shareholders? *Neth Heart J*. 2007;15:127–128.
10. Viskin S, M Wilde AA, Guevara-Valdivia ME, Daoulah A, Krahn A, Zipes DP, Halkin A, Shivkumar K, Boyle NG, Adler A, et al. Quinidine, a life-saving medication for Brugada syndrome, is inaccessible in many countries. *J. Am. Coll. Cardiol.* 2013;61:2383–2387.
11. Postema PG, Schwartz PJ, Arbelo E, Bannenberg WJ, Behr ER, Belhassen B, Brugada J, Brugada P, John Camm A, Casado-Arroyo R, et al. Continued misuse of orphan drug legislation: a life-threatening risk for mexiletine. *Eur. Heart J.* 2020;41:614–617.
12. Wirtz VJ, Hogerzeil HV, Gray AL, Bigdeli M, de Joncheere CP, Ewen MA, Gyansa-Lutterodt M, Jing S, Luiza VL, Mbindyo RM, et al. Essential medicines for universal health coverage. *Lancet*. 2017;389:403–476.
13. van den Berg S, van der Wel V, de Visser SJ, Stunnenberg BC, Timmers L, van der Ree MH, Postema PG, Hollak CEM. Cost-Based Price Calculation of Mexiletine for Nondystrophic Myotonia. *Value Health*. 2021;24:925–929.
14. Cardiac Arrhythmia Suppression Trial (CAST) Investigators. Preliminary report: effect of encainide and flecainide on mortality in a randomized trial of arrhythmia suppression after myocardial infarction. *N.Engl.J Med.* 1989;321:406–412.
15. Waldo AL, Camm AJ, deRuyter H, Friedman PL, MacNeil DJ, Pauls JF, Pitt B, Pratt CM, Schwartz PJ, Veltri EP. Effect of d-sotalol on mortality in patients with left

ventricular dysfunction after recent and remote myocardial infarction. The SWORD Investigators. Survival With Oral d-Sotalol. *Lancet*. 1996;348:7–12.
16. Saljic A, Heijman J, Dobrev D. Recent Advances in Antiarrhythmic Drug Therapy. *Drugs*. 2023;83:1147–1160.
17. Kowey PR, Naccarelli GV. Antiarrhythmic Drug Therapy: Where Do We Go From Here? *Circulation*. 2024;149:801–803.
18. Behr ER, Scrocco C, Wilde AAM, Marijon E, Crotti L, Iliodromitis KE, Remme CA, Kosiuk J, Rudaka I, Brugada GS, et al. Investigation on Sudden Unexpected Death in the Young (SUDY) in Europe: results of the European Heart Rhythm Association Survey. *Europace*. 2022;24:331–339.
19. Eurostat. GDP per capita, consumption per capita and price level indices [Internet]. [cited 2026 Feb 4];Available from: https://ec.europa.eu/eurostat/statistics-explained/index.php?title=GDP_per_capita,_consumption_per_capita_and_price_level_indices
20. Merino JL, Tamargo J, Blomström-Lundqvist C, Boriani G, Crijns HJGM, Dobrev D, Goette A, Hohnloser SH, Naccarelli GV, Reiffel JA, et al. Practical compendium of antiarrhythmic drugs: a clinical consensus statement of the European Heart Rhythm Association of the European Society of Cardiology. *Europace*. 2025;27:euaf076.
21. Svennberg E, Merino JL, Andrade J, Anselmino M, Arbelo E, Boersma E, Boriani G, Breithardt G, Chung M, Chyou J, et al. Transforming atrial fibrillation management by targeting comorbidities and reducing atrial fibrillation burden: the 10th AFNET/EHRA consensus conference. *Europace*. 2025;27:euaf318.
22. Lenarczyk R, Zeppenfeld K, Tfelt-Hansen J, Heinzel FR, Deneke T, Ene E, Meyer C, Wilde A, Arbelo E, Jędrzejczyk-Patej E, et al. Management of patients with an electrical storm or clustered ventricular arrhythmias: a clinical consensus statement of the European Heart Rhythm Association of the ESC-endorsed by the Asia-Pacific Heart Rhythm Society, Heart Rhythm Society, and Latin-American Heart Rhythm Society. *Europace*. 2024;26:euae049.
23. Zeppenfeld K, Tfelt-Hansen J, de Riva M, Winkel BG, Behr ER, Blom NA, Charron P, Corrado D, Dagres N, de Chillou C, et al. 2022 ESC Guidelines for the management of patients with ventricular arrhythmias and the prevention of sudden cardiac death. *Eur Heart J*. 2022;43:3997–4126.
24. Behr ER, Winkel BG, Ensam B, Alfie A, Arbelo E, Berry C, Cerrone M, Conte G, Crotti L, Corcia CMG, et al. The diagnostic role of pharmacological provocation testing in cardiac electrophysiology. A clinical consensus statement of the European Heart Rhythm Association (EHRA) and the European Association of Percutaneous Cardiovascular Interventions (EAPCI) of the ESC, the ESC Working Group on Cardiovascular Pharmacotherapy, the Association of Paediatric and Congenital Cardiology (AEPC), the Paediatric & Congenital Electrophysiology Society (PACES), the Heart Rhythm Society (HRS), the Asian Pacific Heart Rhythm Society (APHRS), and the Latin American Heart Rhythm Society (LAHRS). *Europace*. 2025;27:euaf067.
25. Chockalingam P, Crotti L, Girardengo G, Johnson JN, Harris KM, van der Heijden JF, Hauer RNW, Beckmann BM, Spazzolini C, Rordorf R, et al. Not all beta-blockers are equal in the management of Long QT syndrome types 1 and 2: higher recurrence of events under metoprolol. *J. Am. Coll. Cardiol.* 2012;60:2092–2099.
26. European Medicines Agency. Public information on medicine shortages [Internet]. [cited 2026 Feb 4];Available from: https://www.ema.europa.eu/en/human-regulatory-overview/post-authorisation/medicine-shortages-availability-issues/public-information-medicine-shortages

27. GdziePolLek SP. Z O.O. GdziePolLek (Pharmacies in Poland) [Internet]. [cited 2026 June 1];Available from: https://www.gdziepolek.pl
28. Toniolo M, Muser D, Grilli G, Burelli M, Rebellato L, Daleffe E, Facchin D, Imazio M. Oral procainamide as pharmacological treatment of recurrent and refractory ventricular tachyarrhythmias: A single-center experience. *Heart Rhythm O2*. 2021;2:840–847.
29. Chapman S, Dedet G, Lopert R. Shortages of medicines in OECD countries [Internet]. 2022 [cited 2026 Feb 4]. Available from: https://www.oecd.org/en/publications/shortages-of-medicines-in-oecd-countries_b5d9e15d-en.html
30. Farkowski MM, Scherr D, Boriani G, Kazakiewicz D, Haim M, Huculeci R, Malaczynska-Rajpold K, Petersen SE, Timmis A, Traykov V, et al. Arrhythmia care in ESC member countries: the 2025 ESC-EHRA atlas on heart rhythm disorders. *Europace*. 2025;27:euaf124.
31. De Weerdt E, Simoens S, Hombroeckx L, Casteels M, Huys I. Causes of drug shortages in the legal pharmaceutical framework. *Regul Toxicol Pharmacol*. 2015;71:251–258.
32. Medicines for Europe. Position Paper Medicines shortages [Internet]. 2017 [cited 2026 Mar 16];Available from: https://www.medicinesforeurope.com/docs/20170322_Positionpaper_medicines%20shortages_final.pdf
33. Bogaert P, Bochenek T, Prokop A, Pilc A. A Qualitative Approach to a Better Understanding of the Problems Underlying Drug Shortages, as Viewed from Belgian, French and the European Union’s Perspectives. *PLoS One*. 2015;10:e0125691.
34. Van Oorschot KE, Van Wassenhove LN, Jahre M, Selviaridis K, De Vries H. Drug shortages: A systems view of the current state. *Decision Sciences*. 2022;53:969–984.
35. Casiraghi G, Andres G, Schweitzer F, Verginer L. Supply-chain vulnerabilities in critical medicines: A persistent risk to pharmaceutical security. *Science*. 2025;389:886–888.
36. European Court of Auditors. Special report 19/2025: Critical shortages of medicines – EU measures were of added value, but structural problems remain [Internet]. [cited 2026 Feb 4];Available from: https://www.eca.europa.eu/en/publications?ref=SR-2025-19
37. Postma DJ, De Smet PAGM, Mantel-Teeuwisse AK, Leufkens HGM, Notenboom K. Upstream pharmaceutical supply chains of 10 high-use pharmaceuticals in the Netherlands: a cohort study. *BMJ Open*. 2025;15:e099697.
38. European Medicines Agency. Union list of critical medicines [Internet]. 2026 [cited 2026 Aug 12];Available from: https://www.ema.europa.eu/en/human-regulatory-overview/post-authorisation/medicine-shortages-availability-issues/availability-medicines-during-crises/union-list-critical-medicines
39. Osoro L, Zylla MM, Braunschweig F, Leyva F, Figueras J, Pürerfellner H, Merino JL, Casado-Arroyo R, Boriani G. Challenging the status quo: a scoping review of value-based care models in cardiology and electrophysiology. *Europace*. 2024;26:euae210.

Figure 1.

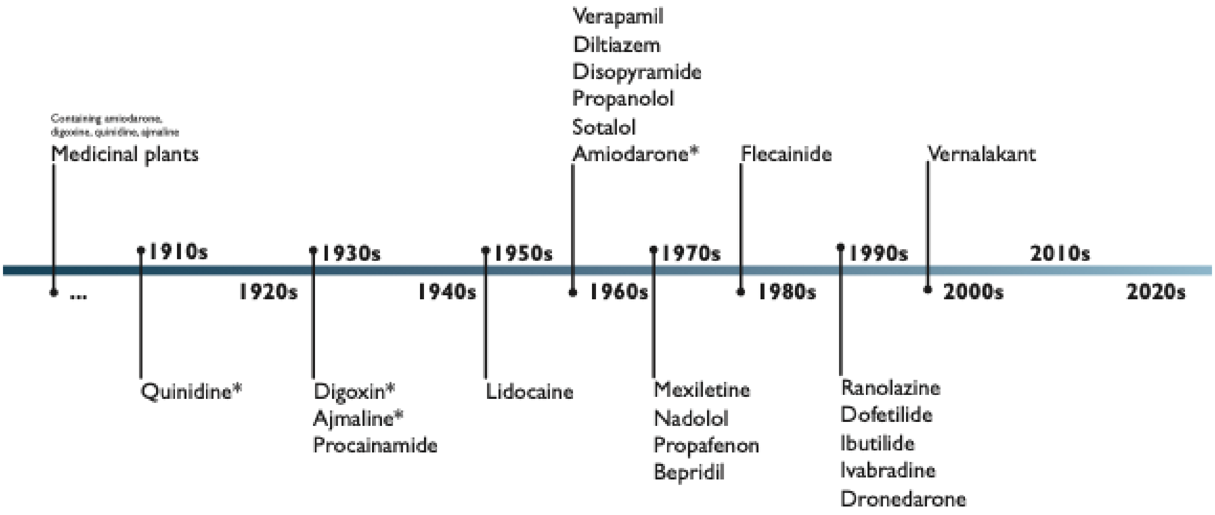

Figure 1. The recognition and development of antiarrhythmic drugs throughout history. Medicinal plants have been used for many centuries while their specific active ingredients were developed in the 20th century (*). Some drugs have been designated as antiarrhythmic drugs many years after their development (e.g. ranolazine).

Figure 2. Reported accessibility of surveyed antiarrhythmic drugs by country grouping among countries with ≥5 respondents.

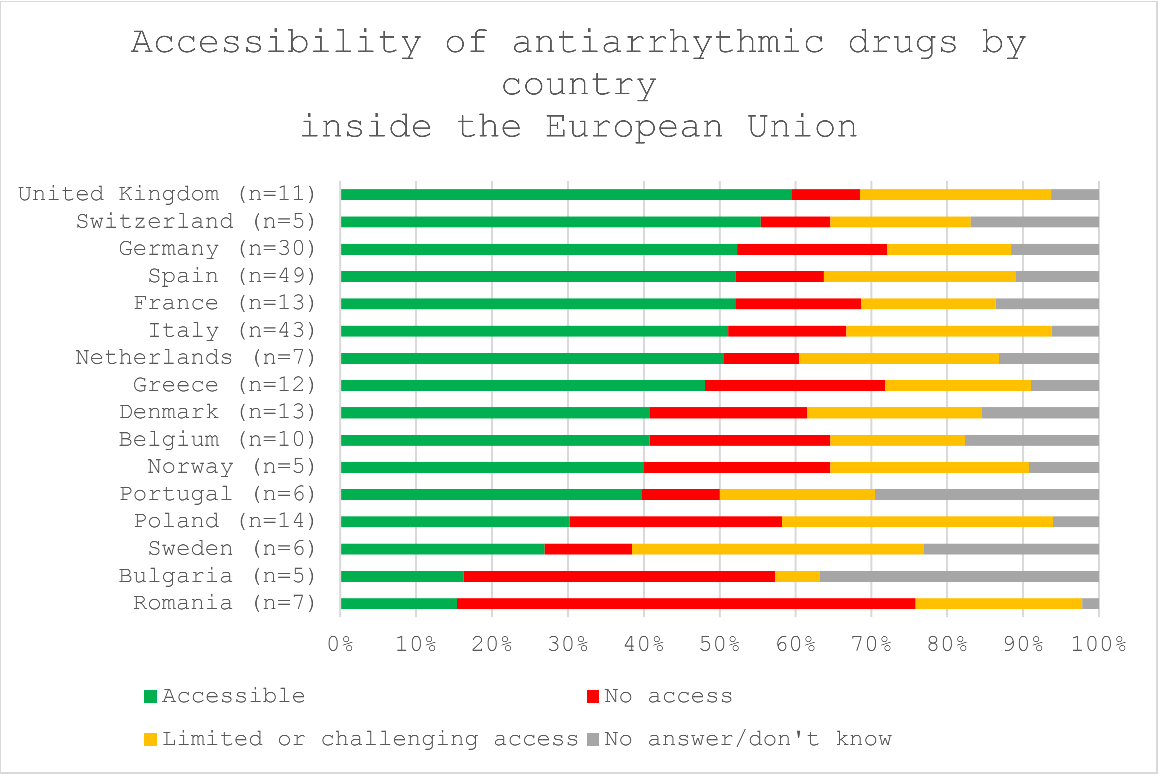

Figure 2A: Reported accessibility of surveyed antiarrhythmic drugs by country within the European Union (plus Norway, Switzerland and the United Kingdom) with ≥5 respondents.

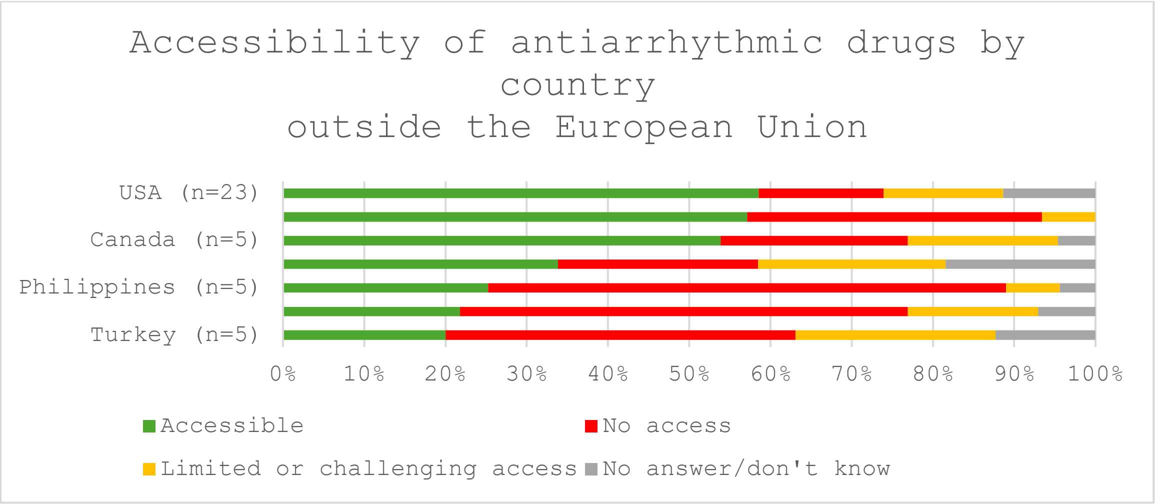

Figure 2B: Accessibility of surveyed antiarrhythmic drugs by country outside the European Union with ≥5 respondents.

Figure 3. Reported accessibility of individual antiarrhythmic drugs, ranked by country grouping among countries with ≥5 respondents.

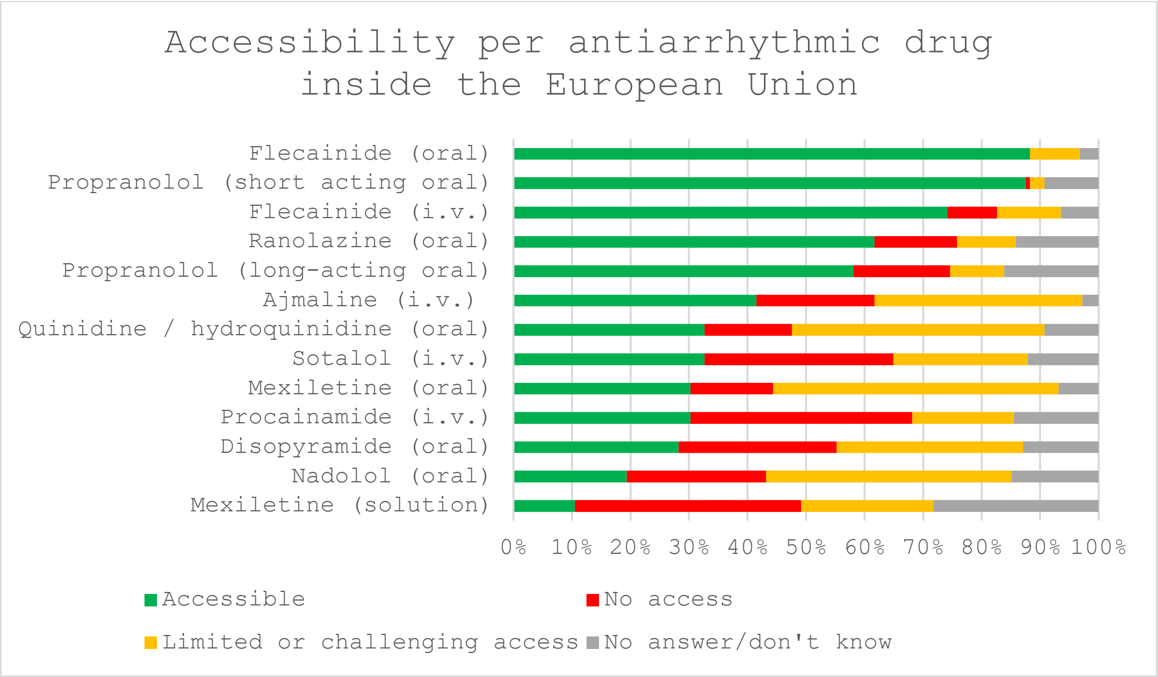

Figure 3A: Accessibility of individual antiarrhythmic drugs (ranked) across countries in the European Union (plus Norway, Switzerland and the United Kingdom) with ≥5 respondents.

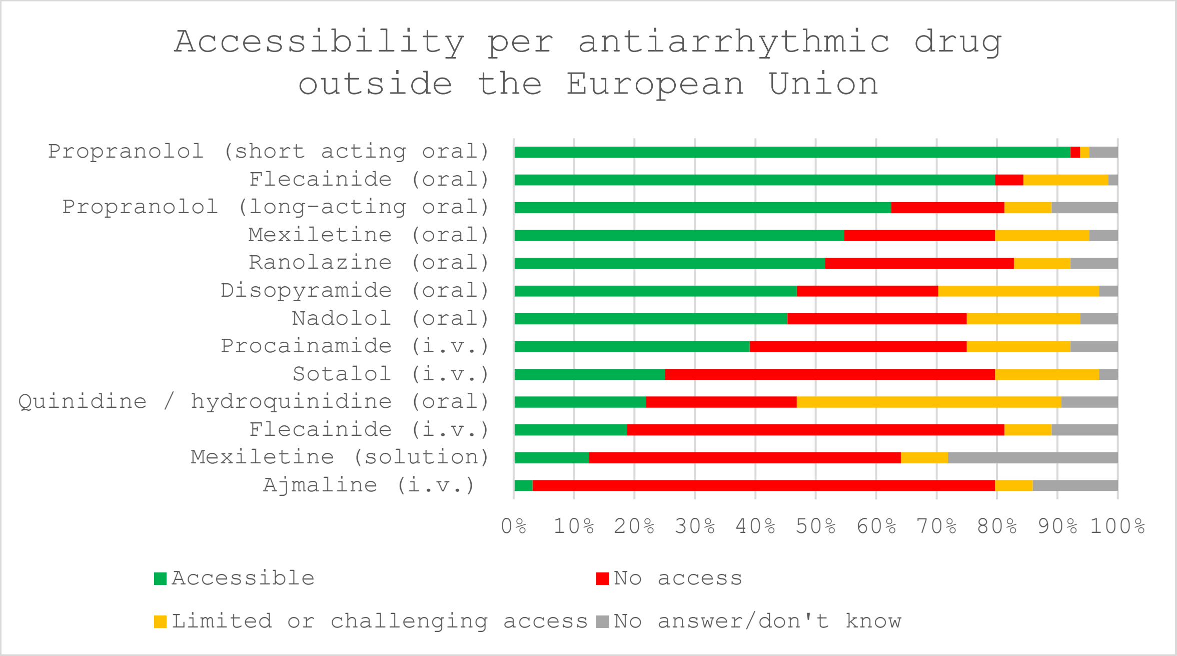

Figure 3B: Accessibility of individual antiarrhythmic drugs (ranked) across countries outside the European Union with ≥5 respondents.

Figure 4. Number of registered products inside the European Union

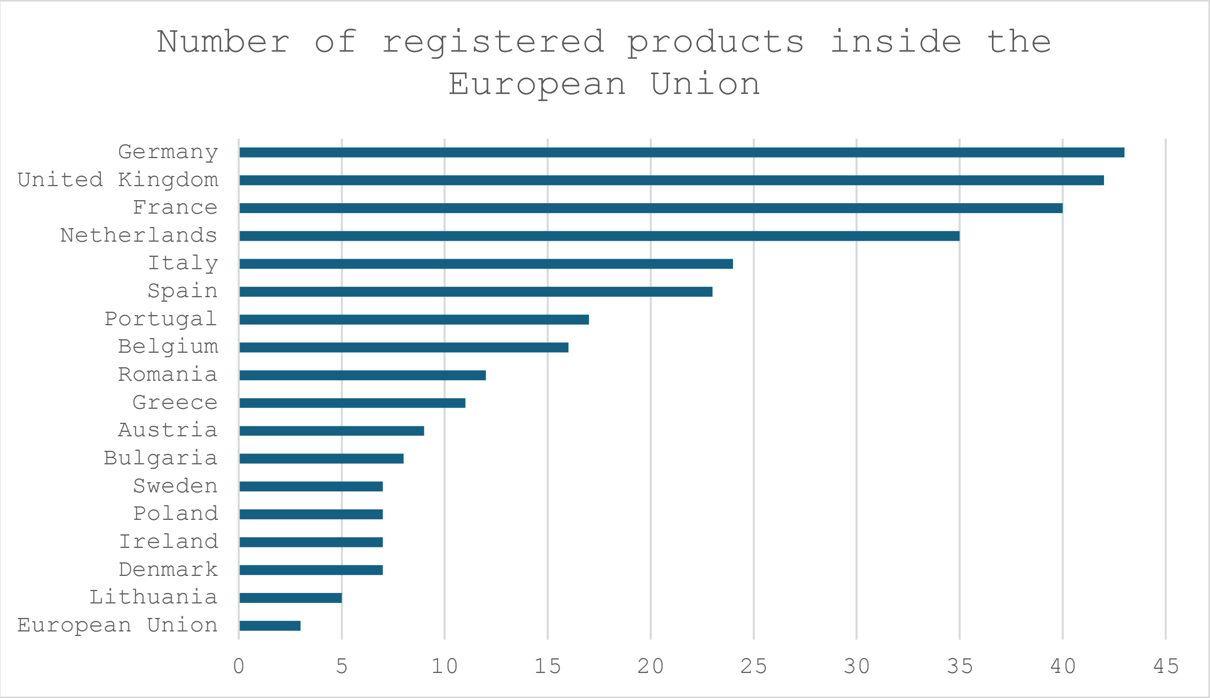

Figure 4: Number of registered products in countries with ≥5 respondents (limited to countries in the European Union, plus the United Kingdom). Note that companies can also register drugs in the 'European Union' in contrast to (separate) country level registration.

Figure 5. Number of registered antiarrhythmic drug products per active ingredient inside the European Union.

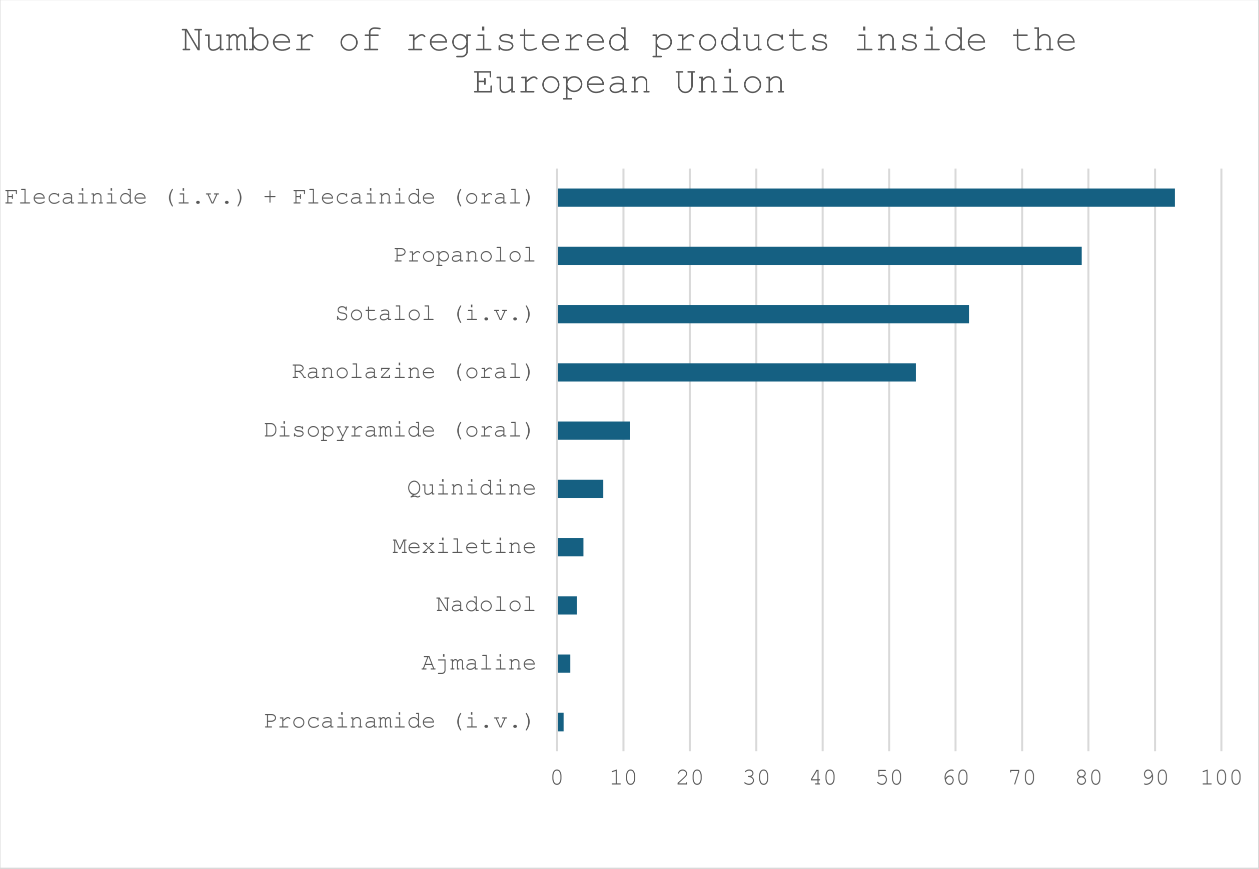

Figure 5: Number of registered antiarrhythmic drug products per active substance (limited to countries in the European Union, plus the United Kingdom).

Supplement figures

Supplemental figure 1. Accessibility of Class antiarrhythmic drugs

Only drugs with low reported accessibility in at least five countries are shown. Each panel displays the modal accessibility category among respondents, reported per country (minimum 3 respondents), color-coded from green (accessible) to red (no access). Countries where at least one third of respondents reported a different category are marked with a white dot pattern on the map and an asterisk (*) in the sidebar. Non-European Union countries are listed in the right-hand sidebar.

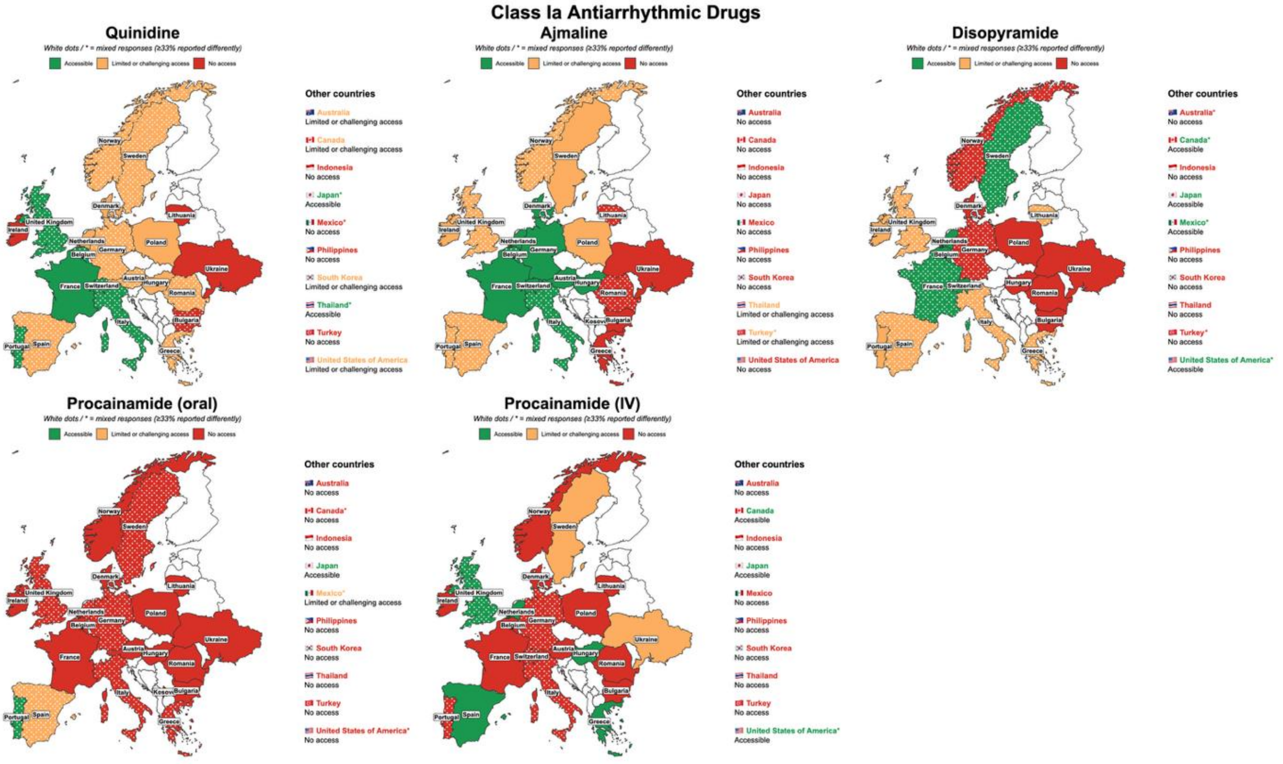

Class Ia Antiarrhythmic Drugs
Quinidine
Ajmaline
Disopyramide
Procainamide (oral)
Procainamide (IV)
Other countries

1

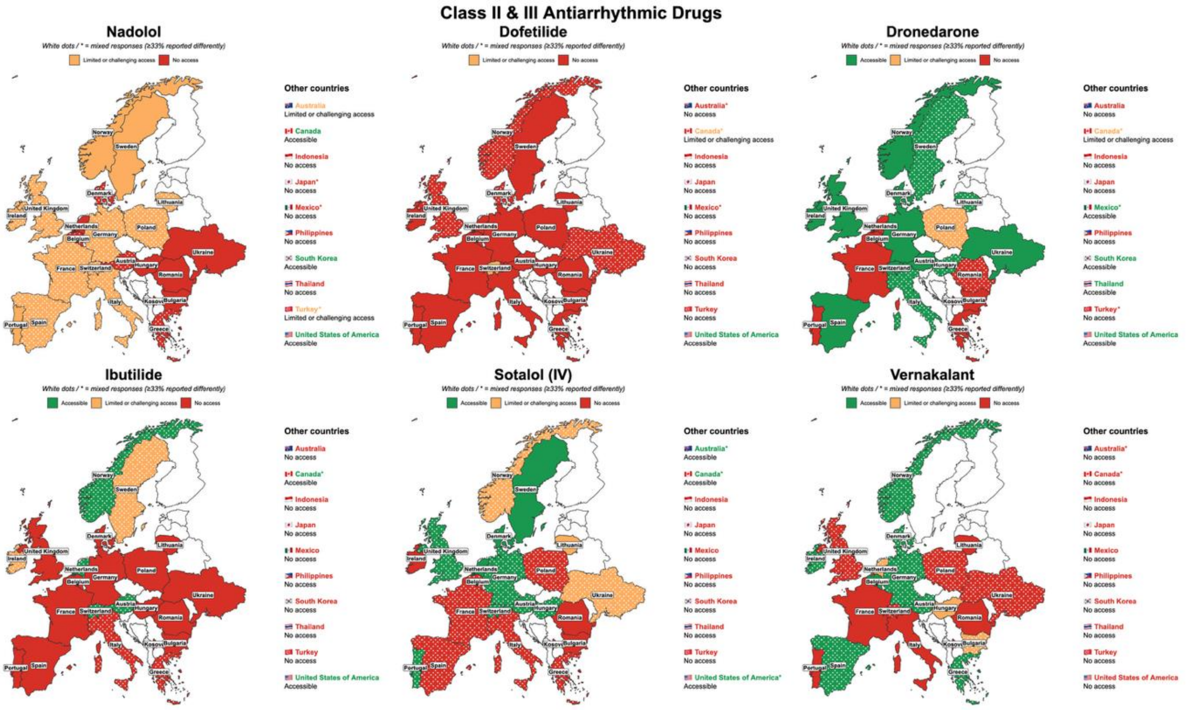

Class II & III Antiarrhythmic Drugs
Nadolol
Dofetilide
Dronedarone
Ibutilide
Sotalol (IV)
Vernakalant
Other countries

2

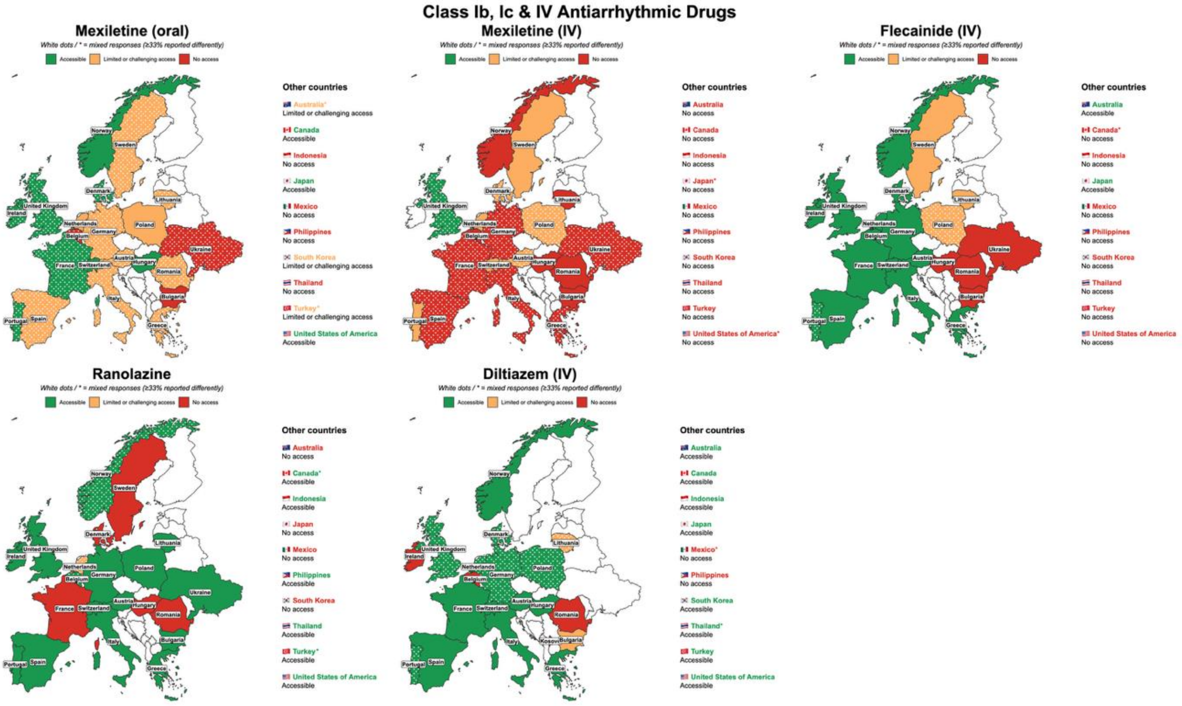
Class Ib, Ic & IV Antiarrhythmic Drugs
Mexiletine (oral)
White dots / * = mixed responses (≥33% reported differently)
Accessible
Limited or challenging access
No access
Other countries
Australia*
Limited or challenging access
Canada
Accessible
Indonesia
No access
Japan
Accessible
Mexico
No access
Philippines
No access
South Korea
Limited or challenging access
Thailand
No access
Turkey*
Limited or challenging access
United States of America
Accessible
Mexiletine (IV)
White dots / * = mixed responses (≥33% reported differently)
Accessible
Limited or challenging access
No access
Other countries
Australia
No access
Canada
No access
Indonesia
No access
Japan*
No access
Mexico
No access
Philippines
No access
South Korea
No access
Thailand
No access
Turkey
No access
United States of America*
No access
Flecainide (IV)
White dots / * = mixed responses (≥33% reported differently)
Accessible
Limited or challenging access
No access
Other countries
Australia
Accessible
Canada*
No access
Indonesia
No access
Japan
Accessible
Mexico
No access
Philippines
No access
South Korea
No access
Thailand
No access
Turkey
No access
United States of America
No access
Ranolazine
White dots / * = mixed responses (≥33% reported differently)
Accessible
Limited or challenging access
No access
Other countries
Australia
No access
Canada*
Accessible
Indonesia
Accessible
Japan
No access
Mexico
No access
Philippines
Accessible
South Korea
No access
Thailand
Accessible
Turkey*
Accessible
United States of America
Accessible
Diltiazem (IV)
White dots / * = mixed responses (≥33% reported differently)
Accessible
Limited or challenging access
No access
Other countries
Australia
Accessible
Canada
Accessible
Indonesia
Accessible
Japan
Accessible
Mexico*
No access
Philippines
No access
South Korea
Accessible
Thailand*
Accessible
Turkey
Accessible
United States of America
Accessible

Supplemental figure 2: Responses per EU country to initial survey.

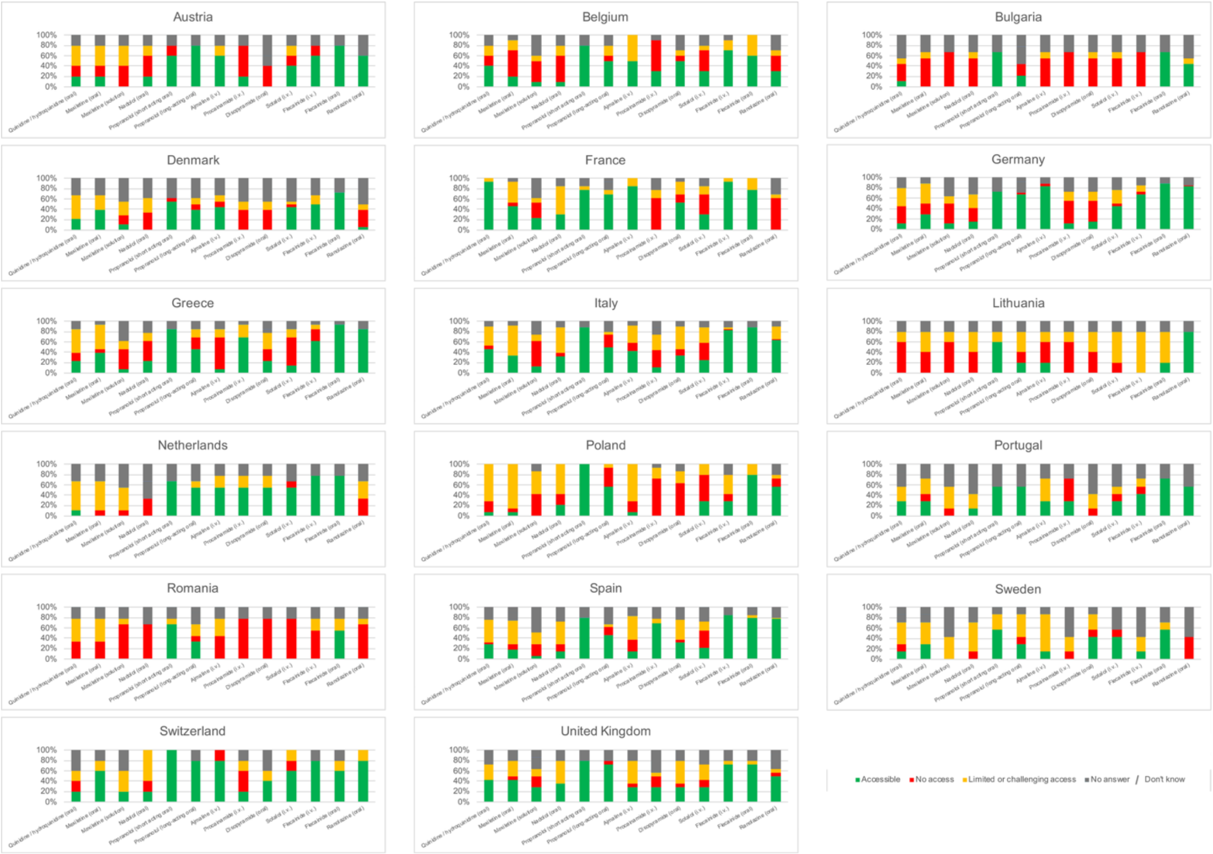

Supplemental Figure 3: Perceived accessibility for all surveyed AAD.

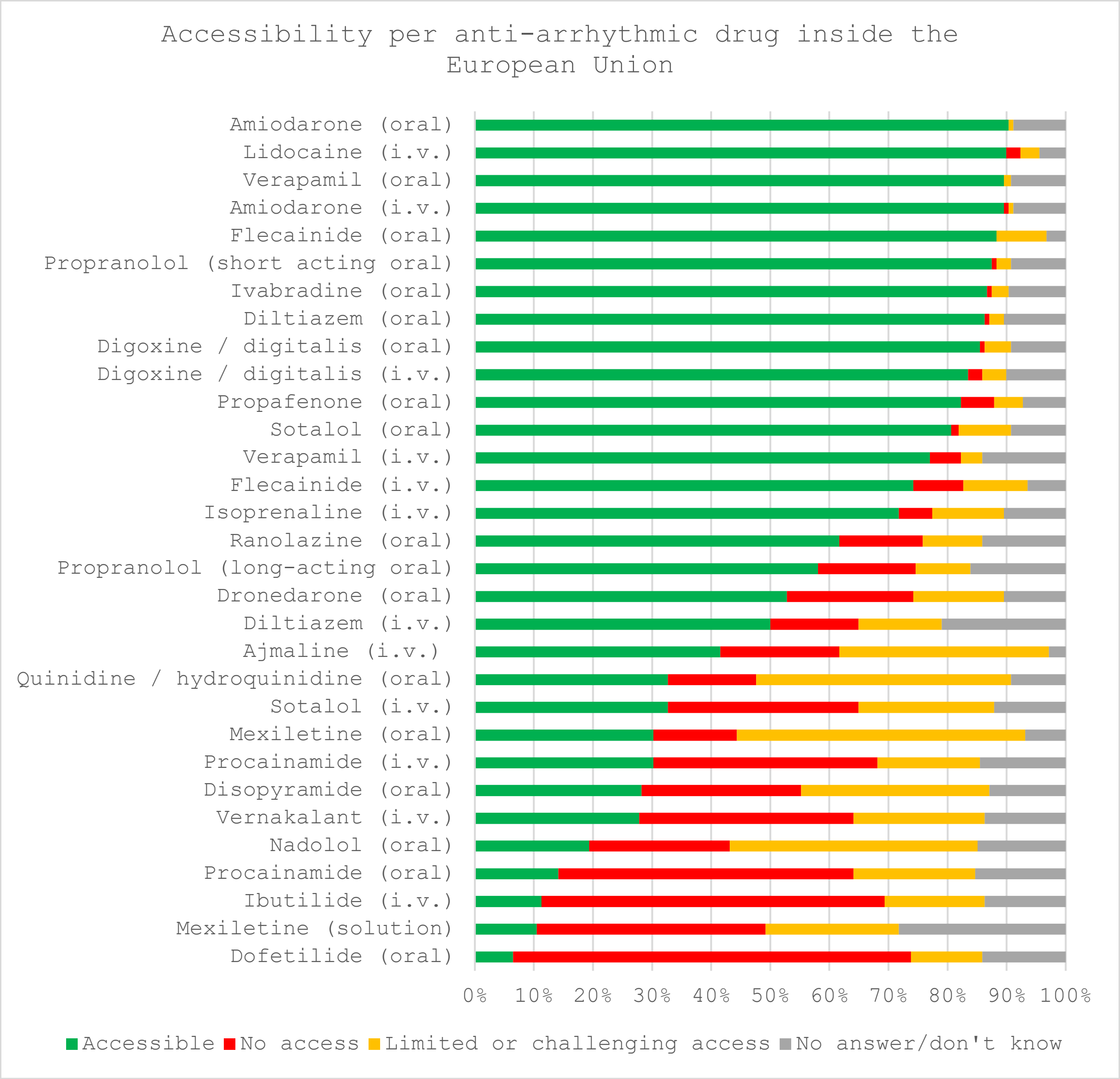

Supplemental Figure 3A: Perceived accessibility for all AAD inside the EU.

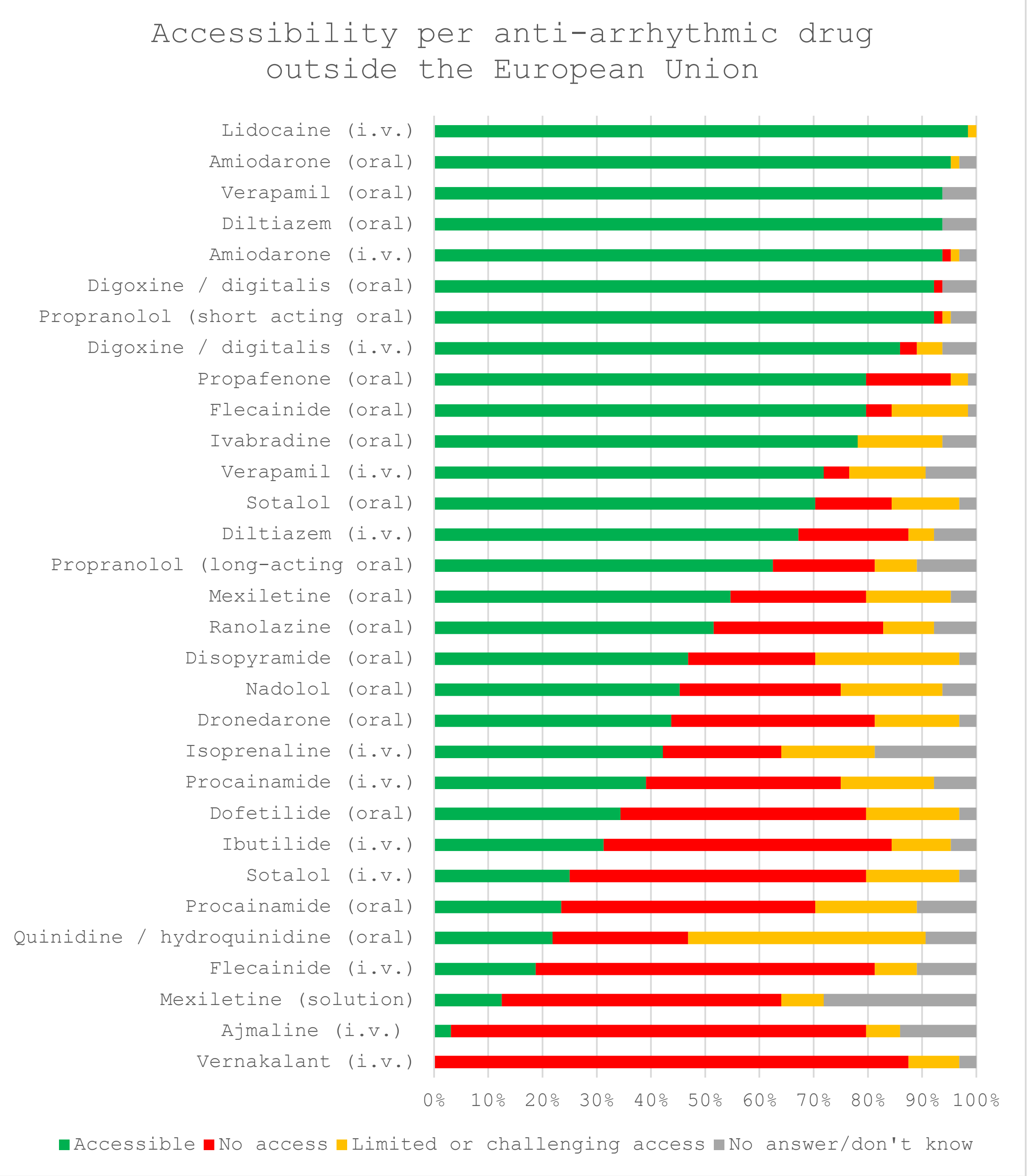

Supplemental Figure 3B: Perceived accessibility for all AAD outside the EU.

Supplemental figure 4. Drug accessibility solutions and barriers considered by the survey responders.

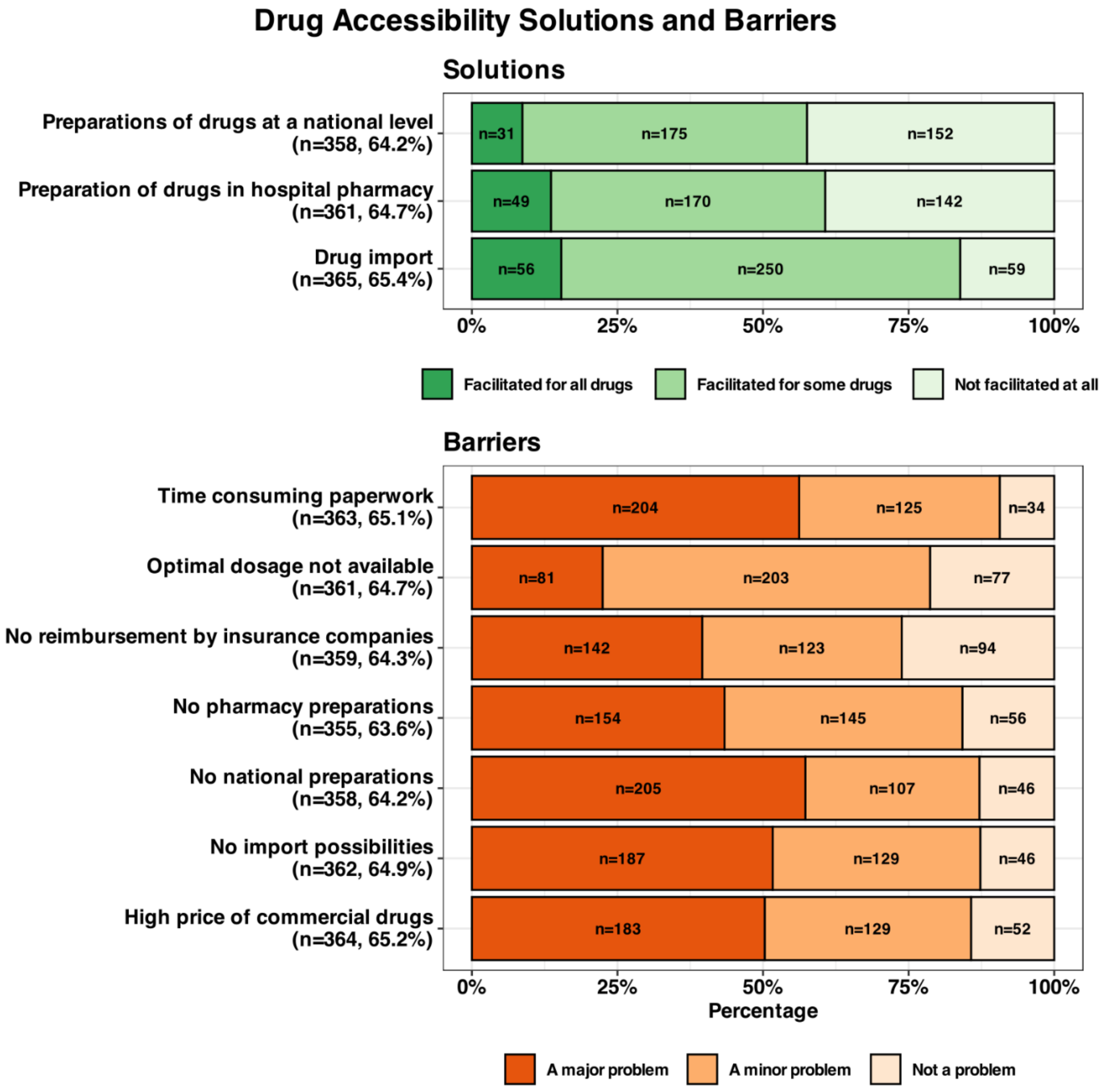

Supplemental figure 5. Distribution of treatment costs of antiarrhythmic drugs per country as indicated by the survey responders.

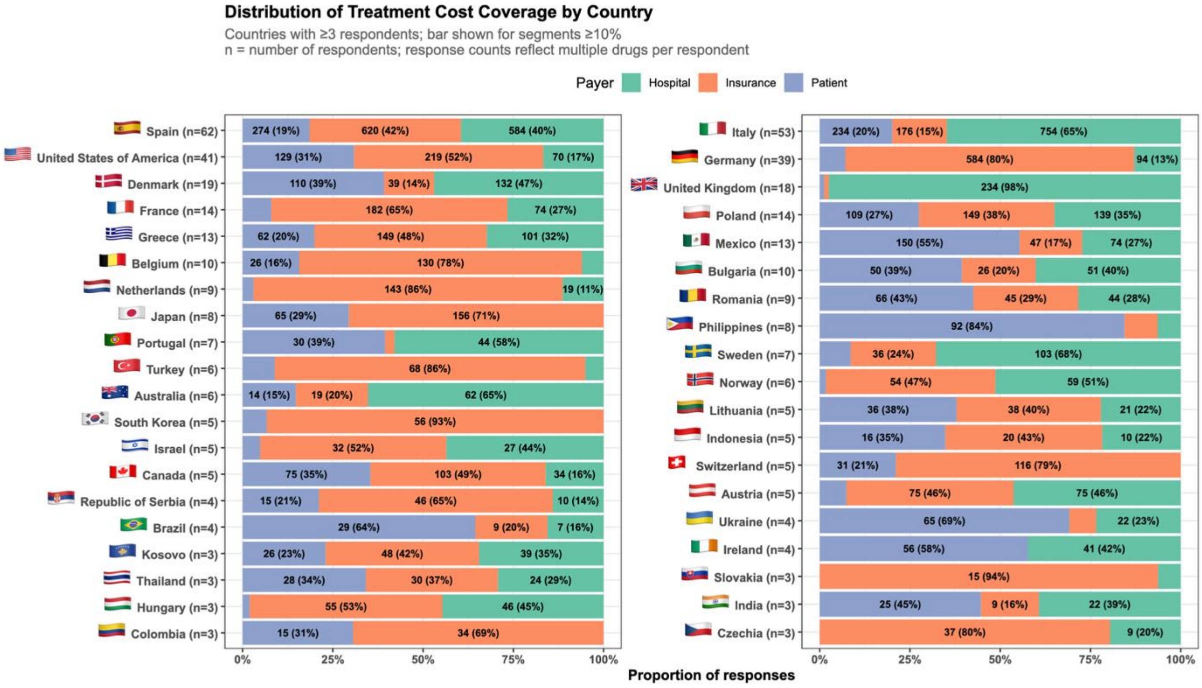

Supplement: Survey distributed to the EHRA members

Survey on accessibility to drugs for arrhythmia

Introduction

1.
GDPR Disclaimer
We will not disclose your identity to any third party.
We comply with the European General Data Protection Regulation (GDPR) 2016/679. Any personal data processed in connection with this survey will be treated confidentially and only used by the ESC for the purposes of market research and not for promotion. Survey results will be kept for a maximum of 48 months for analysis and quality control purposes. We take all reasonable care to prevent any unauthorised access to your personal data. We respect your privacy and your right to access, modify, or remove your personal data. At any time, you can ask to know what personal data is being held. If you have any questions about data protection or require further information, please contact our data protection officer (DPO) at dpo@escardio.org.
You have the right to end your participation in this survey at any time.
Please confirm that you have read the above and agree to participate in this survey.

- ◯ Yes
- ◯ No

Survey on accessibility to drugs for arrhythmia

General Questions

2. Please indicate your gender

- Female
- Male
- Do not want to say
- Other (please specify)

3. What is your age range?

- Younger than 30
- 30-39
- 40-49
- 50-59
- 60-69
- 70 or older

4. In which country is your centre based?

EHRA
European Heart
Rhythm Association

Survey on accessibility to drugs for arrhythmia

General questions on your institution and practice

5. Which of the following best describes your primary workplace?

- ◯ Academic hospital
- ◯ Non-academic hospital
- ◯ Private hospital / clinic
- ◯ Office-based practice
- ◯ Research institution / Research university
- ◯ Other (please specify)

6. Which best describes your profession?

- ◯ Physician – General Cardiology
- ◯ Physician – Sub-specialty in cardiology (heart failure, arrhythmia, imaging, acute, prevention)
- ◯ Physician – Internal Medicine
- ◯ Physician – General Practice
- ◯ Nurse / Pharmacist / Allied health professional
- ◯ Scientist / researcher
- ◯ Other physician
- ◯ Other (please specify)

7. Please state your specialty (multiple answers possible)

- [ ] Cardio-genetics
- [ ] Cardiac Electrophysiology
- [ ] Cardiac Imaging
- [ ] Paediatric Cardiology
- [ ] Geneticist
- [ ] Genetic counsellor
- [ ] General cardiologist
- [ ] Heart Failure cardiologist
- [ ] Other (please specify)

8. What is your career stage?

- [ ] In training
- [ ] Practicing
- [ ] Retired

9. What is your position

- ○ Student
- ○ Resident/Fellow/Intern
- ○ Senior doctor/registrar
- ○ Consultant/Senior consultant
- ○ Head of department
- ○ Other (please specify)

10. Which patient category do you treat?

- [ ] Adult
- [ ] Pediatric
- [ ] Both

11. Do you make treatment decisions for patients with the following conditions?

	Yes	No
Atrial fibrillation / atrial flutter / other supraventricular arrhythmia	○	○
Ventricular arrhythmia	○	○
Rare or heritable arrhythmia syndromes/cardiomyopathies	○	○

12. How often do you make treatment decisions for these conditions ?

	Frequency
Atrial fibrillation / atrial flutter / other supraventricular arrhythmia	
Ventricular arrhythmia	
Rare or heritable arrhythmia syndromes/cardiomyopathies	

Survey on accessibility to drugs for arrhythmia

Questions on access, availability and cost issues

The questions below relate to drugs of interest for the different classes (not for indications). Please answer whether there are access, availability or cost/reimbursement issues with the following drugs in your country (i.e. whether patients/professionals can access them through regular means as they would with other common drugs). Please do provide additional information when appropriate.

13. Are there access or availability issues with the following Class 1A drugs in your country? (i.e. professionals can prescribe and patients can acquire them through regular means as they would with other common drugs)?

	Accessibility
Ajmaline (i.v.)	
Disopyramide (oral)	
Procainamide (oral)	
Procainamide (i.v.)	
Quinidine / hydroquinidine (oral)	

Other (please specify)

14. Are there cost issues with the following Class 1A drugs in your country? (multiple answers possible)

	Insurance pay	Hospital pay	Patient pay	Don't know	N/A / No access
Ajmaline (i.v.)	☐	☐	☐	☐	☐
Disopyramide (oral)	☐	☐	☐	☐	☐
Procainamide (oral)	☐	☐	☐	☐	☐
Procainamide (i.v.)	☐	☐	☐	☐	☐
Quinidine / hydroquinidine (oral)	☐	☐	☐	☐	☐

15. Are there access or availability issues with the following Class 1B drugs in your country? (i.e. professionals can prescribe and patients can acquire them through regular means as they would with other common drugs)?

	Accessibility
Lidocaine (i.v.)	
Mexiletine (oral)	
Mexiletine (solution)	

Other (please specify)

16. Are there cost issues with the following Class 1B drugs in your country? (multiple answers possible)

	Insurance pay	Hospital pay	Patient pay	Don't know	N/A / No access
Lidocaine (i.v.)	☐	☐	☐	☐	☐
Mexiletine (oral)	☐	☐	☐	☐	☐
Mexiletine (solution)	☐	☐	☐	☐	☐

17. Are there access or availability issues with the following Class 1C drugs in your country? (i.e. professionals can prescribe and patients can acquire them through regular means as they would with other common drugs)?

	Accessibility
Flecainide (i.v.)	
Flecainide (oral)	
Propafenone (oral)	

Other (please specify)

18. Are there cost issues with the following Class 1C drugs in your country? (multiple answers possible)

	Insurance pay	Hospital pay	Patient pay	Don't know	N/A / No access
Flecainide (i.v.)	☐	☐	☐	☐	☐
Flecainide (oral)	☐	☐	☐	☐	☐
Propafenone (oral)	☐	☐	☐	☐	☐

19. Are there access or availability issues with the following other Class 1 drugs in your country? (i.e. professionals can prescribe and patients can acquire them through regular means as they would with other common drugs)?

	Accessibility
Ranolazine (oral)	

Other (please specify)

20. Are there cost issues with the following other Class 1 drugs in your country? (multiple answers possible)

	Insurance pay	Hospital pay	Patient pay	Don’t know
Ranolazine (oral)	☐	☐	☐	☐

Survey on accessibility to drugs for arrhythmia

Questions on access, availability and cost issues

21. Are there access or availability issues with the following Class 2 drugs in your country? (i.e. professionals can prescribe and patients can acquire them through regular means as they would with other common drugs)?

	Accessibility
Propranolol (short acting oral)	
Propranolol (long-acting oral)	
Nadolol (oral)	

Other (please specify)

22. Are there cost issues with the following Class 2 drugs in your country? (multiple answers possible)

	Insurance pay	Hospital pay	Patient pay	Don't know	N/A / No access
Propanolol (short acting oral)	☐	☐	☐	☐	☐
Propranolol (long-acting oral)	☐	☐	☐	☐	☐
Nadolol (oral)	☐	☐	☐	☐	☐

23. Are there access or availability issues with the following Class 3 drugs in your country? (i.e. professionals can prescribe and patients can acquire them through regular means as they would with other common drugs)?

	Accessibility
Amiodarone (oral)	
Amiodarone (i.v.)	
Dofetilide (oral)	
Dronedarone (oral)	
Ibutilide (i.v.)	
Sotalol (oral)	
Sotalol (i.v.)	
Vernakalant (i.v.)	

Other (please specify)

24. Are there cost issues with the following Class 3 drugs in your country? (multiple answers possible)

	Insurance pay	Hospital pay	Patient pay	Don't know	N/A / No access
Amiodarone (oral)	☐	☐	☐	☐	☐
Amiodarone (i.v.)	☐	☐	☐	☐	☐
Dofetilide (oral)	☐	☐	☐	☐	☐
Dronedarone (oral)	☐	☐	☐	☐	☐
Ibutilide (i.v.)	☐	☐	☐	☐	☐
Sotalol (oral)	☐	☐	☐	☐	☐
Sotalol (i.v.)	☐	☐	☐	☐	☐
Vernakalant (i.v.)	☐	☐	☐	☐	☐

25. Are there access or availability issues with the following Class 4 drugs in your country? (i.e. professionals can prescribe and patients can acquire them through regular means as they would with other common drugs)?

	Accessibility
Diltiazem (oral)	
Diltiazem (i.v.)	
Verapamil (oral)	
Verapamil (i.v.)	

Other (please specify)

26. Are there cost issues with with the following class 4 drugs in your country?(multiple answers possible)

	Insurance pay	Hospital pay	Patient pay	Don’t know	N/A / No access
Diltiazem (oral)	☐	☐	☐	☐	☐
Diltiazem (i.v.)	☐	☐	☐	☐	☐
Verapamil (oral)	☐	☐	☐	☐	☐
Verapamil (i.v.)	☐	☐	☐	☐	☐

27. Are there access or availability issues with the following other drugs in your country? (i.e. professionals can prescribe and patients can acquire them through regular means as they would with other common drugs)?

	Accessibility
Isoprenaline (i.v.)	
Digoxin/digitalis (oral)	
Digoxin/digitalis (i.v)	
ivabradine (oral)	

Other (please specify)

28. Are there cost issues with with the following other drugs in your country?(multiple answers possible)

	Insurance pay	Hospital pay	Patient pay	Don’t know	N/A / No access
Isoprenaline (i.v.)	☐	☐	☐	☐	☐
Digoxin/digitalis (oral)	☐	☐	☐	☐	☐
Digoxin/digitalis (i.v.)	☐	☐	☐	☐	☐
ivabradine (oral)	☐	☐	☐	☐	☐

EHRA
European Heart
Rhythm Association

Survey on accessibility to drugs for arrhythmia

Questions on access, availability and cost issues

29. Do you know of other drugs for arrhythmia with limited or challenging access in your hospital or country? (please provide generic and/or brand name of drug and access issues)

30. How are accessiblity issues addressed/facilitated at present in your hospital / country? (multiple answers allowed)

	Facilitated for all drugs	Facilitated for some drugs	Not facilitated at all
Drug import is possible/available	○	○	○
Preparation of drugs in hospital pharmacy	○	○	○
Preparations of drugs at a national level	○	○	○

Other (please specify the issues you experience)

31. As to drug accessibility, which of the following items are a major problem, minor problem, or not a problem at all?

	A major problem	A minor problem	Not a problem
No import possibilities	○	○	○
No pharmacy preparations	○	○	○
No national preparations	○	○	○
Time consuming	○	○	○
Optimal dosage not available	○	○	○
paperwork			
(Extremely) high price of commercial drugs	○	○	○
No reimbursement by insurance companies	○	○	○

Other (please specify)

32. If you agree to be contacted and if clarifications are needed/helpful please provide an email address
