# Supplementary material for: Essential antiarrhythmic drug accessibility worldwide: a multi-survey study and comprehensive evaluation of access and supply challenges
Source: Europace. 2026 Sep 2;28(9):euag220. doi: 10.1093/europace/euag220 (PMC13554807; doi:10.1093/europace/euag220)
Supplement: Supplementary file 1 [file euag220_supplementary_data.zip › SurveyMonkey_515413266.pdf]

EHRA
European Heart
Rhythm Association

Survey on accessibility to drugs for arrhythmia

Introduction

1.
GDPR Disclaimer
We will not disclose your identity to any third party.
We comply with the European General Data Protection Regulation (GDPR) 2016/679. Any personal data processed in connection with this survey will be treated confidentially and only used by the ESC for the purposes of market research and not for promotion. Survey results will be kept for a maximum of 48 months for analysis and quality control purposes. We take all reasonable care to prevent any unauthorised access to your personal data. We respect your privacy and your right to access, modify, or remove your personal data. At any time, you can ask to know what personal data is being held. If you have any questions about data protection or require further information, please contact our data protection officer (DPO) at dpo@escardio.org.
You have the right to end your participation in this survey at any time.
Please confirm that you have read the above and agree to participate in this survey.

- ◯ Yes
- ◯ No

EHRA
European Heart
Rhythm Association

Survey on accessibility to drugs for arrhythmia

General Questions

2. Please indicate your gender

- ◯ Female
- ◯ Male
- ◯ Do not want to say
- ◯ Other (please specify)

3. What is your age range?

- ◯ Younger than 30
- ◯ 30-39
- ◯ 40-49
- ◯ 50-59
- ◯ 60-69
- ◯ 70 or older

4. In which country is your centre based?

EHRA
European Heart
Rhythm Association

Survey on accessibility to drugs for arrhythmia

General questions on your institution and practice

5. Which of the following best describes your primary workplace?

- ◯ Academic hospital
- ◯ Non-academic hospital
- ◯ Private hospital / clinic
- ◯ Office-based practice
- ◯ Research institution / Research university
- ◯ Other (please specify)

6. Which best describes your profession?

- ◯ Physician – General Cardiology
- ◯ Physician – Sub-specialty in cardiology (heart failure, arrhythmia, imaging, acute, prevention)
- ◯ Physician – Internal Medicine
- ◯ Physician – General Practice
- ◯ Nurse / Pharmacist / Allied health professional
- ◯ Scientist / researcher
- ◯ Other physician
- ◯ Other (please specify)

7. Please state your specialty (multiple answers possible)

- [ ] Cardio-genetics
- [ ] Cardiac Electrophysiology
- [ ] Cardiac Imaging
- [ ] Paediatric Cardiology
- [ ] Geneticist
- [ ] Genetic counsellor
- [ ] General cardiologist
- [ ] Heart Failure cardiologist
- [ ] Other (please specify)

8. What is your career stage?

- [ ] In training
- [ ] Practicing
- [ ] Retired

9. What is your position

- ○ Student
- ○ Resident/Fellow/Intern
- ○ Senior doctor/registrar
- ○ Consultant/Senior consultant
- ○ Head of department
- ○ Other (please specify)

10. Which patient category do you treat?

- [ ] Adult
- [ ] Pediatric
- [ ] Both

11. Do you make treatment decisions for patients with the following conditions?

	Yes	No
Atrial fibrillation / atrial flutter / other supraventricular arrhythmia	○	○
Ventricular arrhythmia	○	○
Rare or heritable arrhythmia syndromes/cardiomyopathies	○	○

12. How often do you make treatment decisions for these conditions ?

	Frequency
Atrial fibrillation / atrial flutter / other supraventricular arrhythmia	
Ventricular arrhythmia	
Rare or heritable arrhythmia syndromes/cardiomyopathies	

EHRA
European Heart
Rhythm Association

Survey on accessibility to drugs for arrhythmia

Questions on access, availability and cost issues

The questions below relate to drugs of interest for the different classes (not for indications). Please answer whether there are access, availability or cost/reimbursement issues with the following drugs in your country (i.e. whether patients/professionals can access them through regular means as they would with other common drugs). Please do provide additional information when appropriate.

13. Are there access or availability issues with the following Class 1A drugs in your country? (i.e. professionals can prescribe and patients can acquire them through regular means as they would with other common drugs)?

	Accessibility
Ajmaline (i.v.)	
Disopyramide (oral)	
Procainamide (oral)	
Procainamide (i.v.)	
Quinidine / hydroquinidine (oral)	

Other (please specify)

14. Are there cost issues with the following Class 1A drugs in your country? (multiple answers possible)

	Insurance pay	Hospital pay	Patient pay	Don’t know	N/A / No access
Ajmaline (i.v.)	☐	☐	☐	☐	☐
Disopyramide (oral)	☐	☐	☐	☐	☐
Procainamide (oral)	☐	☐	☐	☐	☐
Procainamide (i.v.)	☐	☐	☐	☐	☐
Quinidine / hydroquinidine (oral)	☐	☐	☐	☐	☐

15. Are there access or availability issues with the following Class 1B drugs in your country? (i.e. professionals can prescribe and patients can acquire them through regular means as they would with other common drugs)?

	Accessibility
Lidocaine (i.v.)	
Mexiletine (oral)	
Mexiletine (solution)	

Other (please specify)

16. Are there cost issues with the following Class 1B drugs in your country? (multiple answers possible)

	Insurance pay	Hospital pay	Patient pay	Don’t know	N/A / No access
Lidocaine (i.v.)	☐	☐	☐	☐	☐
Mexiletine (oral)	☐	☐	☐	☐	☐
Mexiletine (solution)	☐	☐	☐	☐	☐

17. Are there access or availability issues with the following Class 1C drugs in your country? (i.e. professionals can prescribe and patients can acquire them through regular means as they would with other common drugs)?

	Accessibility
Flecainide (i.v.)	
Flecainide (oral)	
Propafenone (oral)	

Other (please specify)

18. Are there cost issues with the following Class 1C drugs in your country? (multiple answers possible)

	Insurance pay	Hospital pay	Patient pay	Don’t know	N/A / No access
Flecainide (i.v.)	☐	☐	☐	☐	☐
Flecainide (oral)	☐	☐	☐	☐	☐
Propafenone (oral)	☐	☐	☐	☐	☐

19. Are there access or availability issues with the following other Class 1 drugs in your country? (i.e. professionals can prescribe and patients can acquire them through regular means as they would with other common drugs)?

	Accessibility
Ranolazine (oral)	

Other (please specify)

20. Are there cost issues with the following other Class 1 drugs in your country? (multiple answers possible)

	Insurance pay	Hospital pay	Patient pay	Don’t know
Ranolazine (oral)	☐	☐	☐	☐

Survey on accessibility to drugs for arrhythmia

Questions on access, availability and cost issues

21. Are there access or availability issues with the following Class 2 drugs in your country? (i.e. professionals can prescribe and patients can acquire them through regular means as they would with other common drugs)?

	Accessibility
Propranolol (short acting oral)	
Propranolol (long-acting oral)	
Nadolol (oral)	

Other (please specify)

22. Are there cost issues with the following Class 2 drugs in your country? (multiple answers possible)

	Insurance pay	Hospital pay	Patient pay	Don’t know	N/A / No access
Propanolol (short acting oral)	☐	☐	☐	☐	☐
Propranolol (long-acting oral)	☐	☐	☐	☐	☐
Nadolol (oral)	☐	☐	☐	☐	☐

23. Are there access or availability issues with the following Class 3 drugs in your country? (i.e. professionals can prescribe and patients can acquire them through regular means as they would with other common drugs)?

	Accessibility
Amiodarone (oral)	
Amiodarone (i.v.)	
Dofetilide (oral)	
Dronedarone (oral)	
Ibutilide (i.v.)	
Sotalol (oral)	
Sotalol (i.v.)	
Vernakalant (i.v.)	

Other (please specify)

24. Are there cost issues with the following Class 3 drugs in your country? (multiple answers possible)

	Insurance pay	Hospital pay	Patient pay	Don't know	N/A / No access
Amiodarone (oral)	☐	☐	☐	☐	☐
Amiodarone (i.v.)	☐	☐	☐	☐	☐
Dofetilide (oral)	☐	☐	☐	☐	☐
Dronedarone (oral)	☐	☐	☐	☐	☐
Ibutilide (i.v.)	☐	☐	☐	☐	☐
Sotalol (oral)	☐	☐	☐	☐	☐
Sotalol (i.v.)	☐	☐	☐	☐	☐
Vernakalant (i.v.)	☐	☐	☐	☐	☐

25. Are there access or availability issues with the following Class 4 drugs in your country? (i.e. professionals can prescribe and patients can acquire them through regular means as they would with other common drugs)?

	Accessibility
Diltiazem (oral)	
Diltiazem (i.v.)	
Verapamil (oral)	
Verapamil (i.v.)	

Other (please specify)

26. Are there cost issues with with the following class 4 drugs in your country?(multiple answers possible)

	Insurance pay	Hospital pay	Patient pay	Don't know	N/A / No access
Diltiazem (oral)	☐	☐	☐	☐	☐
Diltiazem (i.v.)	☐	☐	☐	☐	☐
Verapamil (oral)	☐	☐	☐	☐	☐
Verapamil (i.v.)	☐	☐	☐	☐	☐

27. Are there access or availability issues with the following other drugs in your country? (i.e. professionals can prescribe and patients can acquire them through regular means as they would with other common drugs)?

	Accessibility
Isoprenaline (i.v.)	
Digoxin/digitalis (oral)	
Digoxin/digitalis (i.v)	
ivabradine (oral)	

Other (please specify)

28. Are there cost issues with with the following other drugs in your country?(multiple answers possible)

	Insurance pay	Hospital pay	Patient pay	Don’t know	N/A / No access
Isoprenaline (i.v.)	☐	☐	☐	☐	☐
Digoxin/digitalis (oral)	☐	☐	☐	☐	☐
Digoxin/digitalis (i.v.)	☐	☐	☐	☐	☐
ivabradine (oral)	☐	☐	☐	☐	☐

Survey on accessibility to drugs for arrhythmia

Questions on access, availability and cost issues

29. Do you know of other drugs for arrhythmia with limited or challenging access in your hospital or country? (please provide generic and/or brand name of drug and access issues)

30. How are accessiblity issues addressed/facilitated at present in your hospital / country? (multiple answers allowed)

	Facilitated for all drugs	Facilitated for some drugs	Not facilitated at all
Drug import is possible/available	○	○	○
Preparation of drugs in hospital pharmacy	○	○	○
Preparations of drugs at a national level	○	○	○

Other (please specify the issues you experience)

31. As to drug accessibility, which of the following items are a major problem, minor problem, or not a problem at all?

	A major problem	A minor problem	Not a problem
No import possibilities	○	○	○
No pharmacy preparations	○	○	○
No national preparations	○	○	○
Time consuming paperwork	○	○	○
Optimal dosage not available	○	○	○
(Extremely) high price of commercial drugs	○	○	○
No reimbursement by insurance companies	○	○	○

Other (please specify)

32. If you agree to be contacted and if clarifications are needed/helpful please provide an email address
